# Leptin as a Potential Modifier of Neuroinflammation: Contrasting Roles in Alzheimer’s Disease and Multiple Sclerosis

**DOI:** 10.3390/ijms27010168

**Published:** 2025-12-23

**Authors:** Naghmeh Abbasi Kasbi, Barbara Elena Stopschinski, Alanna Gabrielle Polyak, Agastya Reddy Malladi, Navid Manouchehri, Philipp E. Scherer, Olaf Stuve

**Affiliations:** 1 Department of Neurology, University of Texas Southwestern Medical Center, Dallas, TX 75390, USA; 2 Peter O’Donnell Jr. Brain Institute, University of Texas Southwestern Medical Center, Dallas, TX 75390, USA; 3 Center for Alzheimer’s and Neurodegenerative Diseases (CAND), University of Texas Southwestern Medical Center, Dallas, TX 75390, USA; 4 Department of Biological Sciences, Dartmouth College, Hanover, NH 03755, USA; 5 Touchstone Diabetes Center, University of Texas Southwestern Medical Center, Dallas, TX 75390, USA; 6 Neurology Section, VA North Texas Health Care System, Dallas, TX 75216, USA

**Keywords:** leptin, neuroinflammatory diseases, neurodegenerative diseases, multiple sclerosis, Alzheimer’s disease, immune regulation, leptin resistance

## Abstract

The neuroendocrine and immune systems interact bidirectionally through shared ligands and receptors during inflammation, thereby regulating immune responses. Leptin, primarily known for its role in energy metabolism and appetite regulation, also modulates neuroinflammatory pathways. Its receptors are widely expressed on immune cells and contribute to immune mechanisms implicated in the pathogenesis of neuroinflammatory disorders such as multiple sclerosis (MS) and Alzheimer’s disease (AD). This review highlights recent advances in understanding leptin’s role in immune regulation, with a focus on its impact on MS and AD. A comprehensive literature review was conducted until October 2025, using PubMed, Google Scholar, and Scopus to identify studies investigating leptin in neuroinflammatory conditions, particularly MS and AD. Leptin exerts broad immunomodulatory effects by activating T cells, dendritic cells, and microglia, and promoting their proliferation and phagocytosis. Its elevation enhances Th1 and Th17 responses, drives pro-inflammatory macrophage phenotype polarization, and suppresses regulatory T cell and Th2 responses, immune pathways involved in MS. Peripheral leptin levels are increased in MS, especially during disease exacerbations. In contrast, in AD, they are typically reduced, particularly in patients with normal body mass index (BMI), where their decline contributes to amyloid-β and tau pathology. These divergent patterns position leptin as a bidirectional regulator at the intersection of immunity and neurodegeneration. Additionally, its protective or detrimental effects likely depend on whether it acts under physiological conditions or in the context of obesity-induced leptin resistance. Elevated leptin levels in obesity exacerbate inflammation and diminish its neuroprotective effects. In conclusion, leptin is elevated in MS patients but downregulated in AD, reflecting its bidirectional effects. In leptin resistance, peripheral proinflammatory signaling is maintained while central leptin signaling is restricted, thereby potentially promoting autoimmunity in MS and limiting neuroprotection in AD. Further mechanistic and longitudinal studies are needed to clarify the relationship between leptin dysregulation, leptin resistance, neuroinflammatory and neurodegenerative diseases.

## 1. Introduction

Leptin is a non-glycosylated peptide hormone encoded by the *LEP* gene, predominantly synthesized and secreted by adipocytes [1]. It plays a pivotal role in energy homeostasis and metabolic regulation [2]. While leptin’s role in metabolism is well-characterized, emerging evidence highlights leptin as a regulator of the immune system, with receptors broadly expressed on immune cells [2,3,4].

Recent studies show that leptin contributes to neuroinflammatory pathways by modulating the Th1/Th2 balance and promoting the secretion of proinflammatory cytokines, including tumor necrosis factor (TNF)-α, interleukin (IL)-2, IL-6, and interferon (IFN)-γ, in response to various stimuli [5]. Beyond T cell regulation, leptin exerts broad immunomodulatory effects by influencing macrophages and neutrophil activity and enhancing the expression of proinflammatory mediators that play a central role in the pathogenesis of neuroinflammatory disorders, including multiple sclerosis (MS) [6].

The pathogenic mechanisms underlying MS and Alzheimer’s disease (AD) remain incompletely understood. While both diseases involve chronic neuroinflammation and neurodegeneration, their primary pathogenic drivers differ, with MS being largely characterized by T- and B-cell–mediated autoimmune responses [7] and AD being more closely associated with microglial activation and neurodegenerative cascades [8]. Investigations have shown that circulating leptin levels increase during inflammation, underscoring its potential role in immune responses. Elevated leptin concentrations have been associated with increased risk of MS [9]. In contrast, in AD, leptin levels are typically reduced, particularly in individuals with normal body mass index (BMI), where its decline is associated with amyloid β (Aβ) and tau pathology and impaired neuroprotection [10,11]. In the context of autoimmunity, it is likely not physiological leptin levels, which may even be beneficial in conditions such as AD, but rather persistently elevated leptin levels that may confer risk. Chronic hyperleptinemia in conditions such as obesity can induce leptin resistance, disrupt normal signaling, and sustain a proinflammatory state.

A deeper understanding of leptin’s signaling mechanisms in neuroinflammation, particularly its role in immune regulation, may facilitate the identification of novel therapeutic targets. This review aims to expand current knowledge of leptin’s immunomodulatory functions, elucidate its effects on both innate and adaptive immune responses, and clarify how leptin influences the pathophysiology of neuroinflammatory disorders, with a particular focus on MS and AD.

## 2. Leptin History and Biology

The concept of a circulating adiposity signal emerged from studies in the mid-20th century. Obese (*ob*/*ob*) mice, first identified in 1949 at the Jackson Laboratory, presented with severe hyperphagia and obesity due to a recessive mutation later identified as *ob*. These mice lacked the circulating factor regulating satiety [12,13]. In parallel, Gordon Kennedy hypothesized feedback regulation of energy stores via hypothalamic mechanisms in the 1950s [14].

Hervey’s 1959 experiments also provided experimental evidence for a blood-circulating satiety factor influencing hypothalamic feeding centers [15]. Building on these findings, Douglas L. Coleman’s work in the 1960s demonstrated that *ob*/*ob* mice were deficient in the circulating satiety factor. In contrast, obese *db*/*db* mice harbored mutations in the leptin receptor, leading to leptin insensitivity despite elevated leptin levels [16]. This paradigm was confirmed in 1994 by Jeffrey Friedman and colleagues, who cloned and characterized leptin as the adipocyte-derived hormone correcting the ob phenotype [2].

### 2.1. Leptin and Leptin Receptor Structure

Leptin is a 16 kDa peptide hormone encoded by the *LEP* gene located on chromosome 7q31.3 [1]. It is a four-helix bundle that consists of 146 amino acids. Structurally, leptin features a compact four-helix bundle (α1–α4) arranged in an up-and-down topology, stabilized by a disulfide bond linking the C-terminal tail to the α3–α4 loop [2,17].

Leptin is primarily synthesized and secreted by adipocytes, mostly white adipose tissue, and functions as a central regulator of energy homeostasis, appetite, and metabolic processes [18,19]. Adipocytes are the primary source of circulating leptin, but low levels are also secreted by other tissues, including the placenta, gastrointestinal tract, skeletal muscle, mammary epithelium, brain, and pituitary gland [20,21,22,23,24]. The elevated leptin levels in the neonatal pituitary suggest a role in maintaining circulating leptin during early life, when fat stores are not yet well developed [24]. Additionally, the detection of leptin messenger RNA (mRNA) in the brain does not necessarily indicate active protein synthesis or secretion. Reported levels of putative leptin transcripts and protein in neural tissues are extremely low, making it difficult to exclude potential cross-contamination, uptake of circulating leptin, or traces of adjacent adipose tissue. The broad tissue distribution of leptin-expressing sites suggests functions beyond energy homeostasis, encompassing roles in reproductive biology, gastrointestinal physiology, and immune regulation via binding to the leptin receptor (LepR) [25,26,27].

The LepR, also called the obesity receptor (ObR), is a class I cytokine receptor encoded by the *LEPR* gene on chromosome 1 [28]. The LepR belongs to the glycoprotein 130 family of cytokine receptors. Receptor-cloning experiments demonstrated that alternative mRNA splicing and post-translational processing result in different LepR isoforms that share an identical extracellular ligand-binding domain but differ at the carboxy terminus [28]. LepR exists in 6 different isoforms: the full-length signaling long isoform (ObRb), the soluble isoform (ObRe) lacking both transmembrane and intracellular domains, and the short isoforms (ObRa, ObRc, ObRd, ObRf) that possess only short intracellular domains [29,30,31,32].

The long isoform of the leptin receptor, ObRb, includes an intact intracellular domain essential for signal transduction and mediates leptin’s effects on metabolism, appetite, and neuroendocrine functions [33]. ObRb is not consistently expressed throughout the brain but mainly expressed in hypothalamic nuclei, including arcuate nucleus, ventromedial hypothalamus, dorsomedial hypothalamus, and lateral hypothalamic area [34]. It is also expressed in the hippocampus, cerebellum, cerebral cortex, and brainstem [35,36]. Beyond the nervous system, ObRb is present in peripheral tissues such as pancreatic islets, liver, adrenal glands, gonads, heart, kidneys, and muscle [37,38,39,40,41]. ObRb is also expressed on immune cells, including macrophages, natural killer (NK) cells, microglia, B cells, and T cells, enabling leptin to directly modulate their activation, cytokine release, and survival [42]. Like other class I cytokine receptors, ObRb signals through Janus kinase 2 (JAK2) and downstream signal transducer and activator of transcription (STAT) proteins, mainly STAT3, to regulate gene expression [43].

One of the LepR isoforms is the soluble secreted leptin receptor, ObRe, which is mainly produced by hepatic shedding of the membrane-bound receptor. The circulating isoform is a binding protein that stabilizes leptin in plasma, regulates its half-life, and modulates the proportion of leptin available for binding to membrane-bound LepR. Accordingly, soluble leptin receptor is a significant determinant of leptin sensitivity in vivo, and its measurement has been proposed as an index of leptin resistance in metabolic disease [44,45,46].

Short isoforms are more widely expressed in peripheral tissues and have truncated cytoplasmic tails with partial signaling capability [47]. These short isoforms regulate leptin bioavailability, including its transport across the blood–brain barrier (BBB). ObRa is highly expressed on brain microvascular endothelial cells and on choroid plexus (ChP) epithelial cells. These cells form the BBB and the blood-cerebrospinal fluid barrier (BCSFB), respectively. At these sites, ObRa supports receptor-mediated transport of leptin from the blood into the cerebrospinal fluid (CSF) and brain parenchyma [48].

### 2.2. Leptin Signaling

Leptin biology is primarily initiated by its binding to the leptin receptor and the activation of downstream intracellular signaling pathways [49]. At the molecular level, leptin activates JAK2 through dimerization of its long receptor isoform, ObRb [33]. Activated JAK2 phosphorylates specific tyrosine residues within ObRb, including Tyr985, Tyr1077, and Tyr1138. Among these, phosphorylation of Tyr1138 is pivotal, as it facilitates high-affinity recruitment and activation of STAT3, initiating a prototypical transcriptional program first described in hypothalamic neurons [50]. The central role of leptin on appetite and energy balance is mediated by phosphorylated STAT3 translocating to the nucleus, where it seems possible that STAT3 inhibits agouti-related protein (AGRP) neurons and induces anorexigenic neuropeptides such as pro-opiomelanocortin (POMC). Specific deletion of STAT3 in neurons leads to profound obesity in mice [51]. A recently identified population of hypothalamic neurons expressing the zinc-finger transcription factor basonuclin-2 (BNC2) has been shown to respond directly to leptin signaling. These BNC2 neurons become activated upon leptin stimulation. Once activated, they rapidly suppress appetite by exerting direct inhibitory input onto AGRP neurons, key orexigenic neurons that typically drive feeding behavior [52].

The expression of suppressor of cytokine signaling 3 (SOCS3) acts as a negative feedback regulator of leptin signaling by binding to JAK2, inhibiting its activity. Mice lacking SOCS3 in the brain or specifically in POMC neurons exhibit enhanced leptin sensitivity and are resistant to diet-induced obesity [53,54]. Similarly, protein tyrosine phosphatase 1B (PTP1B) and T-cell protein tyrosine phosphatase (TCPTP) attenuate leptin signaling by dephosphorylating key tyrosine-phosphorylated intermediates. PTP1B directly dephosphorylates JAK2, and PTP1B-deficient mice display reduced food intake, increased leptin sensitivity, and resistance to diet-induced obesity [55]. Similarly, TCPTP targets STAT3 for dephosphorylation, and deletion of TCPTP results in lower body weight, increased energy expenditure, and augmented leptin responsiveness compared with controls [56]. Conversely, mice deficient in cyclic AMP-dependent protein kinase A (PKA) exhibit enhanced leptin sensitivity due to a prolonged duration of hypothalamic JAK2–STAT3 signaling [57].

Adenosine monophosphate-activated protein kinase (AMPK) is an important enzyme that helps cells sense and balance their energy levels. In the hypothalamus, AMPK activity is inhibited by leptin, feeding, and high glucose. Leptin-induced AMPK inhibition increases malonyl-CoA and long-chain fatty acyl-CoA levels, leading to reduced food intake and suppressed hepatic glucose output [58]. Functionally, AMPK activation in the medio-basal hypothalamus drives feeding, whereas its inhibition reduces appetite. Mice lacking AMPK in POMC neurons become obese due to hyperphagia and lower energy expenditure. In contrast, deletion in AGRP neurons produces a lean phenotype [59], indicating cell-type-specific roles in energy balance. Phosphorylation of the AMPK α2 subunit at Ser491 by p70 S6 kinase (p70 S6K) inhibits AMPK activation [60].

In the hypothalamus, leptin triggers the activation of the insulin receptor substrate (IRS) and phosphatidylinositol 3-kinase (PI3K) signaling cascade [61]. IRS proteins act as adaptor molecules that transmit signals from the leptin receptor to downstream enzymes. Activation of PI3K converts PIP2 to PIP3, triggering a cascade that regulates appetite and metabolism. Mice lacking IRS or PI3K show increased food intake, reduced energy expenditure, and resistance to leptin [62,63]. Downstream of PI3K, leptin stimulates protein kinase B (Akt) phosphorylation at Thr308 and Ser473, which then activates the mammalian target of rapamycin (mTOR) pathway. mTOR senses nutrient and energy status to promote protein synthesis and cell growth while reducing autophagy. Leptin increases mTOR and S6 kinase activity, while the mTOR inhibitor rapamycin blunts leptin’s anorectic effects [64].

### 2.3. Leptin Resistance

Leptin resistance refers to a state in which leptin levels are elevated, but the brain’s response to its appetite-suppressing (anorexigenic) effects and other biological effects are blunted due to reduced central leptin sensitivity [65]. Leptin resistance can broadly be categorized into several forms, including high-fat diet-induced, leptin-induced, inflammation-induced, seasonal, pregnancy- or lactation-associated, and endoplasmic reticulum (ER) stress-induced leptin resistance. Multiple mechanisms contribute to this state, including a disrupted leptin transport across the BBB, leptin signaling, overexpression of SOCS3, and alterations in the leptin receptor.

As stated above, leptin is secreted predominantly by white adipose tissue, and its circulating levels correlate directly with fat mass; consequently, obesity is associated with chronically elevated leptin concentrations. Rather than inducing satiety, sustained hyperleptinemia disrupts downstream signaling through ObRb, resulting in impaired appetite regulation and metabolic imbalance [66]. This paradoxical state promotes further weight gain and enhances chronic low-grade inflammation.

Chronic hyperleptinemia has been demonstrated to impair leptin responsiveness by saturating downstream JAK2-STAT3 signaling, a key pathway for appetite suppression [67]. Partial reduction of circulating leptin in diet-induced obese (DIO) mice improved hypothalamic leptin sensitivity, prevented high-fat diet-induced obesity, and corrected glucose dysregulation [68], underscoring hyperleptinemia as both a driver and consequence of leptin resistance. As stated above, leptin signaling is regulated by a negative feedback loop mediated by SOCS3, which usually constrains its activity. However, elevated leptin levels in obesity increased SOCS3 expression, which in turn inhibits JAK2–STAT3 activation and further reduces leptin signaling and sensitivity [65]. Additionally, lipid metabolite accumulation enhances the expression of protein kinase C theta (PKC-θ), thereby attenuating the PI3K pathway of leptin signaling [69]. Moreover, chronic mTOR activation in POMC neurons during aging can paradoxically lead to hyperphagic obesity, suggesting that the effect of mTOR depends on the dosage and duration of its activation [70]. Recent evidence indicates that enhanced mTOR signaling within POMC neurons is necessary and sufficient for the development of leptin resistance in DIO mice [71].

Sustained hyperleptinemia in obesity appears to downregulate LepR expression, likely through receptor saturation and feedback inhibition. Consistently, DIO mice show decreased hypothalamic LepR mRNA despite elevated leptin levels [72], suggesting that diminished receptor responsiveness contributes to obesity-associated leptin resistance. Additionally, early investigations demonstrated that obesity and high-fat diet intake markedly reduce specific leptin transporters, including the expression of the short leptin receptor isoform, ObRa, in brain endothelial cells [73]. It has been suggested that ObRa down-regulation limits leptin entry into the CNS, thereby reducing central leptin signaling, diminishing satiety responses, promoting hyperphagia, and accelerating weight gain [48]. However, recent studies in DIO mice and obese humans set intact leptin transport kinetics in specific settings, but calorie restriction or glucagon-like peptide-1 receptor agonist (GLP-1RA) treatment enhanced LepR expression [73]. The resistance is now known to be a multifactorial process, including defective receptor signaling, endoplasmic reticulum (ER) stress, and overexpression of inhibitory controllers like SOCS3, and possibly to some extent a transport failure across the BBB. Ultimately, these mechanisms contribute to persistently elevated leptin levels in both the periphery and the CNS.

Serum leptin measurement alone is insufficient for diagnosing leptin resistance. The focus cannot be solely on circulating leptin concentrations, as these are influenced not only by *LEP* gene expression but also by leptin protein clearance and the presence and functional activity of its receptors. To date, no standardized diagnostic criteria have been established. Recently, several studies have proposed using both high BMI and high circulating leptin level as an indirect indicator of leptin resistance [74,75]. This approach assumes that individuals with higher BMI may exhibit a disproportionate increase in leptin levels, reflecting a state of resistance. While this ratio provides a convenient, non-invasive tool, it should be interpreted with caution and additional assessments. Nonetheless, in the absence of standardized methods, it remains one of the few practical approaches available for estimating leptin resistance [76,77].

### 2.4. Leptin Transport Mechanisms Across the Blood–Brain Barrier

Studies have provided evidence that leptin crosses the BBB via a saturable, receptor-mediated mechanism. Multiple studies support a key role for ObRa in regulating the translocation of leptin through the BBB [78,79]. It was shown that, in mice, following intravenous administration of radiolabeled leptin, brain influx was ~20-fold higher than that of tagged albumin, consistent with active rather than passive diffusion. Autoradiographic analyses further demonstrated leptin uptake in the ChP, arcuate nucleus, and median eminence, with no detectable efflux, supporting leptin’s unidirectional entry [80]. However, another study using intracerebroventricular leptin administration showed that leptin gradually exits the CNS through CSF reabsorption, enters the circulation, and becomes detectable in the serum [81]. This delivery system is physiologically significant: intravenous leptin preferentially localizes to the arcuate nucleus, a key hypothalamic feeding center, whereas intracerebroventricular administration fails to replicate this distribution, highlighting the BBB’s dual role as both a barrier and a regulated transport pathway [81].

Recent evidence proposed that low-density lipoprotein receptor-related protein 2 (LRP2), megalin, might mediate leptin transport across the BBB; however, experimental findings demonstrate that leptin signaling and uptake occur independently of this receptor, as its inhibition or deletion fails to affect leptin transcytosis or metabolic regulation [82].

Data implicate the ChP as an entry route into the CSF. Genetic models also verified this role, since conditional ablation of the leptin receptors in brain endothelial and ChP epithelial cells of mice showed highly impaired leptin uptake, became hyperphagic and obese on a high-fat diet, and showed alterations in reward-related feeding circuits with no effect on homeostatic feeding, indicating barrier-localized LepR as critical regulators of energy balance [83]. Human studies demonstrated that leptin transport across the BBB and binding to its receptors are temperature-, saturation-, and competition-dependent manner [84]. Modern imaging with fluorescently labeled leptin and light-sheet microscopy in mice demonstrated leptin enrichment in the ChP and circumventricular regions, including the median eminence and subfornical organ. Further, it showed that DIO does not abolish leptin accumulation in them. Additionally, calorie restriction or glucagon-like peptide-1 receptor agonist (GLP-1RA) treatment enhanced LepR expression in the ChP and increased hypothalamic leptin content, indicating plasticity of barrier-mediated transport under metabolic interventions [73].

The role of the ChP in leptin transport across the BCSF barrier, mediated by high ObRa expression, is vital because the ChP also functions as an immunologically active interface that facilitates the transfer of cytokines, immune cells, and signaling molecules between the blood and CSF [83]. In mouse model of MS, ChP epithelial cells increase adhesion molecules including intercellular adhesion molecule (ICAM-1), vascular cell adhesion molecule (VCAM-1) and chemokines that induce the influx of pro-inflammatory lymphocytes into the ventricular space: a function probably aided by the established capacity of leptin to increase Th1 and Th17 differentiation and suppress regulatory T (Treg) cells function [85]. In MS, histopathological studies have shown ChP epithelial hypertrophy, major histocompatibility complex (MHC) class II upregulation, and lymphocytic infiltration around perivascular lesions. Notably, leptin receptors have been detected in inflamed ChP epithelium, raising the possibility that leptin signaling in this region, particularly under conditions of obesity, may associate with CNS immune activation [86].

Collectively, leptin signaling at the ChP not only facilitates hormone transport into the CSF but may also modulate the immune activity of ChP by enhancing the expression of adhesion molecules and chemokines that recruit peripheral immune cells into the CNS. Through this mechanism, leptin can regulate the trafficking of pro-inflammatory T cells, monocytes, and other immune effectors across the ChP, thereby linking metabolic status to neuroimmune responses [87]. Taken together, leptin’s evolution from a satiety hormone to an immune-metabolic regulator provides a framework for its direct effects on immune cell biology.

## 3. Leptin and Immune Cells

**Figure 1:**
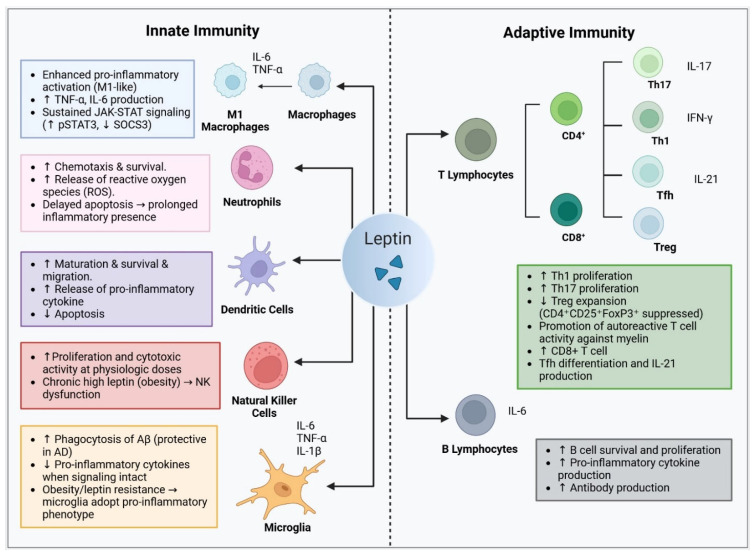
**Leptin regulates innate and adaptive immunity.** (**Left panel**): Leptin drives macrophages toward a proinflammatory state, increases neutrophil and DC survival and ROS release, enhances NK cell activity (but causes dysfunction with chronic high levels), and shifts microglia toward an inflammatory profile in obesity or leptin resistance. (**Right panel**): In adaptive immunity, leptin promotes Th1 and Th17 responses, suppresses Tregs, and supports B cell survival, cytokine release, and antibody production (Schematic created with BioRender 201).

Leptin receptors are broadly expressed on different subsets of innate and adaptive immune cells, through which leptin mediates its immunomodulatory effects on the immune system [25,88,89]. To further elucidate the role of leptin in immune responses, one study assessed the expression of ObRb at both mRNA and protein levels in freshly isolated and activated immune cells. They demonstrated that CD4^+^ T cells, B cells, and monocytes all express ObRb mRNA, and that specific activation of each cell population markedly upregulated its mRNA expression [90]. These findings underscore leptin’s potential role in sustaining immune cell function and provide the basis for detailed investigation of its specific effects on individual immune cell populations (Figure 1).

### 3.1. Leptin and Innate Immunity

Leptin is shown to interact with components of the innate immune system, including neutrophils, macrophages, dendritic cells (DCs), NK cells, and specifically, within the CNS, microglia.

#### 3.1.1. Neutrophils

Polymorphonuclear neutrophils are granulocytes essential for innate immunity, responding to the sites of infection, tissue damage, and inflammation [91]. It has been shown that leptin can affect neutrophil migration, activation, and survival [92,93,94].

Leptin has been shown to recruit neutrophils into both the periphery and the CNS, functions as a chemoattractant for neutrophils, promoting their recruitment and antimicrobial activity. Wild-type (WT) bone marrow neutrophils exhibit chemotaxis toward leptin. This response is reduced in LepR Q223R variants. This mutation functionally impairs receptor activity by disrupting STAT3-mediated signal transduction in vitro [95,96] and is impaired in *db*/*db* mice, which show decreased neutrophil trafficking during infection [97,98]. In a study conducted by Souza et al. [92], evaluating the potential of leptin to activate and induce migration of neutrophils, it was demonstrated that intraperitoneal injection of leptin in mice allows for a concentration-dependent influx of neutrophils. Specifically, it was noted that the recruitment was dependent on TNF-α and the chemokine (C-X-C motif) ligand (CXCL)1. Furthermore, neutrophil migration did not occur in TNF-α receptor1 and PI3K-γ knockout mice, indicating the possibly critical roles of TNF-α and PI3K-γ in these signaling pathways. In the experiment, leptin-mediated neutrophil recruitment occurred independently of 5-lipoxygenase (5-LO) and mTOR signaling [92]. It was also shown that following LPS-induced inflammation, leptin-deficient and leptin-resistant mice showed distinctly reduced neutrophil brain infiltration with decreased expression of neutrophil-specific chemokines, including IL-1β and ICAM-1 [93]. Supplementation of leptin in leptin-deficient mice restored the process of neutrophil recruitment, and 48 h food deprivation in WT mice, which decreased leptin levels, limited neutrophil recruitment [93]. Furthermore, in EAE models, the ablation of endothelial leptin signaling significantly reduced CD11b^+^Gr1^+^ neutrophil infiltration into the spinal cord, underscoring leptin’s role in facilitating neutrophil trafficking across the blood-CNS barrier during neuroinflammation [94].

Leptin may also protect against neuroinflammation by stabilizing the BBB and thereby limiting neutrophil-mediated tissue damage. In models of ischemia, intracisternal leptin administration reduced BBB permeability, inhibited matrix metalloproteinase-9 activation, and decreased neutrophil infiltration into the brain [99].

Despite the evidence supporting leptin’s role in modulating neutrophil migration, its role in neutrophil activation is less direct. Human neutrophils express only ObRa receptors, while monocytes express both ObRa and ObRb. Leptin was shown to increase CD11b on neutrophils in whole blood but not in purified neutrophils. Inhibition of TNF-α blocked 71% of leptin’s stimulatory effect on neutrophils, indicating that leptin may induce neutrophil activation peripherally through crosstalk with monocytes [100]. Furthermore, leptin’s effect on neutrophil activation may be concentration dependent. It was shown that normal blood concentrations of leptin (around 250 ng/mL) failed to induce neutrophil activation and chemotaxis in vitro. However, high doses (25,000 ng/mL) were sufficient to induce notable changes in neutrophils, supporting their survival, although chemotaxis was still undetected [101]. These findings suggest that leptin’s effects on neutrophils and other leukocytes may be concentration dependent.

In terms of neutrophil survival, leptin may prolong neutrophil function by delaying apoptosis. It was found that activation of leptin receptors on neutrophils induces the PI3K- and MAPK-dependent signaling cascades. There was a noted inhibition of cytochrome c release, stabilization of mitochondrial membranes, and suppression of caspase-8 and -3 activation. These effects were also found to be concentration dependent [102]. Additionally, leptin stimulates human polymorphonuclear neutrophils to produce ROS in vitro, potentially enhancing bacterial clearance through their oxidative capacity [103].

Leptin’s effects on neutrophils appear to vary depending on physiological and pathological contexts. For instance, during pneumonia and acute lung injury, leptin has been shown to promote neutrophil recruitment [104]. Similarly, in obese patients, hyperleptinemia is associated with enhanced neutrophilic airway inflammation mediated through macrophage-derived CXCL2, highlighting leptin’s role in myeloid crosstalk and Th1-driven immune responses [105]. Conversely, leptin can also exert inhibitory effects on neutrophil function. In end-stage renal disease (ESRD), hyperleptinemia was linked to impaired neutrophil chemotaxis, an effect that was reversible upon leptin depletion [106]. Although leptin does not trigger calcium mobilization, ROS generation, or β2-integrin upregulation, its capacity to desensitize neutrophils may underlie the heightened susceptibility to infection observed in hyperleptinemic states such as ESRD [106]. These findings suggest that leptin may play dual roles in neutrophil biology. Leptin acts as a chemotactic factor that directly induces neutrophil migration but may also inhibit their response to classical chemoattractants.

In conclusion, leptin’s role is multifaceted in the regulation of neutrophils, influencing their migration, activation, and survival. Neutrophil recruitment appears to occur through direct leptin signaling, whereas activation is likely mediated indirectly via monocyte crosstalk. Moreover, leptin’s effects are context-dependent, resulting in diverse outcomes such as amplified recruitment, prolonged survival, or impaired chemotaxis. In neuroinflammatory diseases specifically, leptin regulates neutrophil trafficking across the BBB and can either limit or exacerbate CNS damage.

#### 3.1.2. Macrophages

Macrophages are tissue-resident mononuclear phagocytes of the innate immune system that originate primarily from circulating monocytes [107,108]. Macrophages respond to leptin by adopting a reactive phenotype characterized by increased pro-inflammatory cytokine production and enhanced phagocytic activity. Leptin activates multiple signaling pathways in macrophages, including the JAK/STAT3, PI3K/Akt, and mitogen-activated protein kinase (MAPK; p38 and c-Jun N-terminal kinase (JNK)) cascades [107,109].

Macrophages express ObRb and respond to the concentration of leptin by increasing their proliferation and activation. It was found that human leptin activates circulating human monocytes in vitro in a dose-dependent manner, subsequently increasing the production of proinflammatory cytokines, including TNF-α and IL-6, as well as upregulating activation markers such as CD25, HLA-DR, and CD38, along with CD11b and CD11c markers [107]. Macrophages’ response to leptin was comparable to that induced by endotoxin lipopolysaccharide (LPS) stimulation, further underscoring leptin’s potential to initiate immune activation [107]. Leptin can also modulate macrophage polarization. It was found that in vitro, leptin associates with LPS and IFN-γ to facilitate a pro-inflammatory macrophage phenotype, previously referred to as M1 polarization, characterized by elevated production of cytokines such as IL-6, IL-1β, and TNF-α [105]. Clinically, obese asthma patients showed higher leptin levels and stronger M1 polarization with a positive correlation between serum leptin and pro-inflammatory macrophage phenotype activity [105].

Experimental models of obesity further demonstrate that leptin sensitizes macrophages to LPS, thereby promoting enhanced cytokine release, mitochondrial remodeling, and glycolytic metabolism through mTORC2-dependent pathways [5]. Leptin activates both mTOR complexes in macrophages, inducing phosphorylation of the mTORC1 target eukaryotic initiation factor 4E-binding protein (4EBP1) and the mTORC2 target Akt-Ser473. When combined with LPS, leptin preferentially sustained Akt-Ser473 phosphorylation, while LPS alone primarily activated S6 kinase (mTORC1), with no amplification by leptin. Pretreatment with high-dose rapamycin, which inhibits both mTORC1 and mTORC2, reduced phosphorylation of 4EBP1 and Akt-Ser473, confirming the involvement of both complexes. These results demonstrate that leptin differentially modulates mTORC1 and mTORC2 signaling in the context of LPS stimulation [5]. Using conditional knockouts, the study showed that leptin amplifies inflammatory cytokine production primarily through mTORC2 signaling [5]. Consistently, hyperleptinemia resulting from diet-induced obesity enhances inflammatory macrophage activity, whereas deletion of LepR in these cells reduces systemic inflammation [5].

Beyond its proinflammatory actions, leptin has been shown to support pathogen clearance via macrophages. Leptin deficiency in *ob*/*ob* mice led to increased susceptibility to LPS-induced lethality, in part due to limited induction of IL-1 receptor antagonist and IL-10. The addition of leptin successfully restored cytokine responses and reduced mortality, demonstrating its role in protecting against inflammatory conditions through immunoregulatory functions [110]. Leptin has also been shown to enhance phagocytosis in infectious disease models. In *Leishmania donovani*-infected THP-1 cells and human peripheral blood mononuclear cells (PBMCs), leptin promoted macrophage activation, increased reactive oxygen species (ROS) production, and improved phagolysosome formation [111]. Similarly, in alveolar macrophages lacking LepR, pneumococcal infection models revealed impaired bacterial clearance and defective phagocytosis, further underscoring leptin’s importance in macrophage-driven host defense [112].

Leptin can also influence macrophage function by inducing programmed cell death protein (PD)-1 expression through pathways dependent on mTORC1 signaling and glycolysis [113]. This can act as a negative feedback mechanism, limiting macrophage phagocytosis and diminishing their capacity to activate T cells, impairing tumor immune surveillance. This data suggested that PD-1 contributes to the obesity-cancer link, as obesity-associated inflammatory signals and enhanced glycolysis upregulate PD-1 expression on macrophages via mTORC1, serving as a negative feedback mechanism to restrain glycolysis and inflammatory responses [113].

Within the CNS, leptin signaling in macrophages is associated with a worse CNS autoimmunity course based on data in experimental autoimmune encephalomyelitis (EAE), namely through increased leptin expression by macrophages within the peripheral secondary lymphoid organs [114]. Leptin impacts the CNS through its receptor expression in infiltrating T cells and macrophages during neuroinflammatory disease. In EAE, a mouse model for studying autoimmune disease of the CNS, leptin levels have been shown to surge before disease onset, correlating with increased macrophage and T cell activation. This surge enhances the expression of proinflammatory cytokines, including IL-2, IL-12, IFN-γ, and TNF-α, thereby contributing to the exacerbation of CNS disease [114]. In obesity, hyperleptinemia may cause increased neuroinflammation by facilitating macrophage activation and promoting pro-inflammatory macrophage phenotype as previously mentioned [5,105]. This continued leptin signaling in macrophages likely exacerbates neuronal injury and increases the recruitment of immune cells via the release of cytokines.

Leptin functions as a central immunomodulator of macrophages, driving proinflammatory cytokine production and phagocytosis. These effects are accentuated under hyperleptinemic conditions. Inhibition of LepR or its downstream signaling attenuates this inflammatory activity; however, such interventions must also account for leptin’s essential roles in immune regulation and pathogen clearance [5].

#### 3.1.3. Dendritic Cells

DCs are professional antigen-presenting cells that link innate and adaptive immunity by recognizing pathogen-associated molecular patterns (PAMPs) through pattern recognition receptors and presenting processed antigens to T cells. In chronic inflammation, obesity, and autoimmunity, DC-induced T cell activation is associated with increased levels of leptin [115]. Leptin’s capacity to modulate DC viability, maturation, cytokine secretion, metabolic programming, migration, and T cell polarization establishes it as a pivotal immune-metabolic regulator.

Among the first descriptions of leptin’s involvement in DC biology were experiments with *ob*/*ob* leptin-deficient mice. Bone marrow-derived DCs in these mice exhibited profoundly defective maturation. The surface expression of conventional costimulatory and antigen-presenting molecules such as CD40, CD80, CD86, and MHC-II was reduced compared to WT controls [115]. Immature DCs were functionally impaired, producing less IL-12, TNF-α, and IL-6 compared to WT DCs, while simultaneously producing more transforming growth factor β (TGF-β) [115]. In T cell co-culture assays, these DCs showed limited capacity to induce CD4^+^ T cell proliferation. Adding recombinant leptin restored the functional and phenotypic defects to WT levels, highlighting leptin’s key role in DC differentiation and immune function [115].

At the functional level, leptin fundamentally remodels the human DC cytokine output. In vitro experiments demonstrate that leptin stimulation induces an increase in IL-1β, IL-6, IL-12, TNF-α, and macrophage inflammatory protein (MIP)-1α, but suppresses IL-10 secretion [116]. These alterations induce a pro-inflammatory DC phenotype. Proliferation of CD4^+^ T cells was increased in response to leptin-activated DCs compared to untreated controls. Cytokine environment dysregulation promotes adaptive immune responses towards the Th1 phenotype while suppressing regulatory pathways [116]. The influence of leptin-conditioned DCs on T cell polarization has been elucidated in experiments. Both Th1 and Th17 differentiation is robust in leptin-treated DCs. It was reported that there was an increase in IFN-γ-secreting CD4^+^ T cells and IL-17A-secreting CD4^+^ T cells compared to reactions against untreated DCs [115]. Foxp3^+^ regulatory T cell induction, however, was reduced in leptin-conditioned DCs [115]. These effects are driven by DC-derived IL-12 and IL-6, as well as by leptin-induced STAT3 activation in both DCs and T cells. Overall, this shifts the adaptive immune response away from tolerance and towards inflammation, especially under hyperleptinemic conditions such as obesity or chronic infection [115]. Metabolically conditioned DCs also reciprocally induced pro-inflammatory T cell polarization: in co-culture experiments, leptin-conditioned DCs expanded IL-17A^+^ CD4^+^ T cells. Inhibiting glycolysis with 2-deoxyglucose abolished both DC activation and DC-driven Th17 differentiation [117]. Thus, leptin not only promotes DC maturation and survival but also metabolically conditions them for chronic pro-inflammatory function.

Resting DCs rely on oxidative phosphorylation, but upon activation, their metabolism is reprogrammed toward glycolysis to support antigen presentation and cytokine production. Leptin accelerates this glycolytic shift. In human monocyte-derived DCs, leptin increased glucose uptake, lactate production, and expression of the glycolytic enzyme, hexokinase 2 (HK2) [117]. These metabolic alterations were dependent on STAT3 activation; inhibiting STAT3 reduced HK2 overexpression [117]. Aside from maturation, leptin also has a strong anti-apoptotic effect on DCs. Leptin signaling is required for DCs development. At the pre-diabetic stage, *db*/*db* mice generated significantly fewer bone-marrow-derived DCs than WT controls, and leptin blockade with soluble recombinant leptin receptor (ObR: Fc) similarly suppressed DC generation in WT cultures. This reduction is associated with increased apoptosis and altered expression of B-cell leukemia/lymphoma-2 (Bcl-2) family proteins. LepR-deficient DCs express lower levels of co-stimulatory molecules, produce predominantly Th2-type cytokines, and stimulate allogeneic T cells poorly. PI3K/Akt, STAT3, and inhibitor of nuclear factor-κB alpha (IκB-α) signaling pathways are also reduced. Collectively, these findings indicate that leptin supports DC survival and maturation [118]. In human monocyte-derived DCs, leptin exposure suppressed spontaneous apoptosis and enhanced resistance to oxidative and genotoxic stress, as survival was increased under UVB irradiation in leptin-treated cells [119]. Leptin promoted cell survival through PI3K-Akt activation and subsequent upregulation of the anti-apoptotic proteins Bcl-2 and Bcl-X. Inhibition of PI3K abrogated leptin’s pro-survival effect [119]. These findings emphasize that leptin not only promotes DC maturation but also preserves their viability under stress, thereby prolonging their antigen-presenting function under inflammatory conditions.

Leptin regulates DC migratory behavior, guiding their trafficking from peripheral tissues to lymphoid organs. In mice, leptin enhances immature DC migration by remodeling the actin cytoskeleton and upregulating C-C chemokine receptor (CCR)7 on DCs, inducing chemotaxis in response to gradients of C-C motif chemokine ligand (CCL)19/CCL21 [120,121]. Remarkably, this migratory response was abolished in LepR-deficient DCs, consistent with receptor dependence [118].

Disease models show the physiological significance of leptin-DC interactions. In murine breast cancer models, DCs stimulated with leptin (particularly when combined with LPS) reduced tumor-promoting gene expression, including matrix metalloproteinase 9 (MMP9), CCL5, and vascular endothelial growth factor (VEGF), and decreased IL-6 secretion [122]. Whether similar mechanisms apply in other cancer types remains uncertain. In obesity-related cancers like hepatocellular carcinoma, the chronic hyperleptinemia and inflammatory milieu may instead push DCs toward dysfunctional or immunosuppressive states, but this has yet to be directly demonstrated.

Methodological limitations should be considered, as in vitro studies use widely variable leptin concentrations, ranging from near-physiological (~10–50 ng/mL) to supraphysiological levels (>500 ng/mL), and DC responses across this range are non-linear. Human monocyte-derived DCs, plasmacytoid DCs, and conventional DC subsets respond differently depending on their metabolic state, LepR expression, and the local cytokine setting. Moreover, systemic factors such as obesity, insulin resistance, and sex hormones strongly influence leptin-DC interactions in vivo.

Overall, leptin promotes DC maturation, enhances survival and migration, drives pro-inflammatory cytokine production, reprograms metabolism toward glycolysis, and shifts T cell polarization toward Th1/Th17 lineages. Under physiological conditions, these activities strengthen host defense and enhance vaccine immunogenicity. In contrast, in diseases such as obesity, autoimmunity, and chronic inflammation, they contribute to inappropriate immune activation.

#### 3.1.4. Natural Killer Cells

NK cells are cytotoxic innate lymphoid cells that provide rapid immune surveillance without prior antigen sensitization. In terms of NK cells, leptin is a potent regulator of NK cell functions, including cytotoxic activity, cytokine production, survival, maturation, and activation [123]. Disrupted leptin signaling impairs immune surveillance and contributes to chronic inflammation.

Early studies showed that both murine and human NK cells express LepR and respond to leptin stimulation [124,125]. In vitro, leptin was found to increase NK cell cytotoxicity and cytokine production through STAT3 phosphorylation, transcription of perforin, and release of IFN-γ [125]. Additionally, leptin exposure in human NK cells also upregulated CD69, boosted IFN-γ secretion, and strengthened tumor cell conjugate formation, partly through increased expression of tumor necrosis factor-related apoptosis-inducing ligand (TRAIL) [126]. However, chronic hyperleptinemia has been shown to induce leptin resistance in NK cells. Prolonged exposure led to shortened JAK2 phosphorylation, decreased proliferation, and suppressed IFN-γ and TRAIL production [126,127]. Furthermore, in DIO rats, defects in downstream signaling were found after ObRb binding. The JAK2, Akt, and AMPK pathways were impaired, despite receptor expression, which points to the development of leptin resistance [128].

Obesity and, by extension, dysregulated leptin signaling have been shown to alter NK cell numbers, phenotypes, and effector functions. Across multiple clinical studies in both adults and children, obesity was associated with reduced NK cell frequencies, impaired cytotoxicity, and diminished cytokine secretion, despite evidence of cellular activation [127,129,130,131]. Peripheral NK cells from obese patients displayed elevated activation markers such as CD69 and granzyme B but defective degranulation and reduced tumor-killing capacity [130]. Comparable findings in obese children included decreased NK cell frequencies, heightened mTOR activity, mitochondrial ROS accumulation, and functional exhaustion characterized by impaired proliferation and tumor lysis [132]. In addition to circulating NK cells, obesity promotes their accumulation in visceral adipose tissue, where they produce TNF-α and polarize macrophages toward proinflammatory phenotypes. NK cell depletion in these models attenuated adipose inflammation and improved systemic metabolic homeostasis, identifying NK cells as key instigators of obesity-induced inflammation [133]. Collectively, these studies indicate that obesity is closely linked to NK cell dysfunction, with potential long-term consequences for immune surveillance and chronic inflammation.

Cytokine activation can drive NK cells into a memory-like (ML) state with enhanced effector properties. Exposure to IL-12, IL-15, and IL-18 induces enriched ML NK cells, which are defined by epigenetic and transcriptional reprogramming that reshapes functional responses [134]. Whether leptin signaling contributes to ML NK cell differentiation remains unclear. However, given leptin’s established role in metabolic reprogramming of lymphocytes, leptin resistance in obesity may impair ML NK cell development, thereby suppressing their antiviral and antitumor immunity.

These mechanisms have direct implications for neuroinflammation. First, adipose NK cells initiate systemic inflammation by activating macrophages and producing TNF-α, which may spread into the CNS through cytokine signaling and BBB disruption. Second, exhausted NK cells may fail to clear virally infected or transformed cells, leading to chronic inflammation that contributes to neuroinflammatory processes. Finally, potential impaired ML NK cell differentiation could diminish long-term immunosurveillance and trigger neuroinflammation.

#### 3.1.5. Microglia

Microglia are the yolk sac-derived myeloid cells of the CNS that play an essential role in brain homeostasis [135]. Leptin functions as an immune-metabolic signal for microglia, where LepR activation influences their cytokine release, phagocytosis activity, synaptic interactions, and neurovascular communication [136].

Microglia express both ObRb and ObRa isoforms, with ObRb levels higher than those in astrocytes and neurons [137]. Upon stimulation, leptin induces a dose-dependent production and release of IL-1β along with its endogenous interleukin-1 receptor antagonist (IL-1RA), a process that requires STAT3 signaling yet proceeds independently of caspase-mediated cleavage, indicating a noncanonical maturation route for IL-1β [137]. In amyloid precursor protein (APP)/presenilin-1 (PS1) transgenic mice, a widely used preclinical model of AD, systemic leptin treatment increased Iba1^+^ microglial activation, consistent with a role for leptin in modulating microglial responses in vivo. Additionally, analysis of microglia-associated inflammatory markers revealed elevated levels of IL-1 and IL-6 in the hippocampus of leptin-treated adult mice [136]. In parallel, a 2007 study reported a dose- and time-dependent increase in IL-6 release from BV-2 microglial through the LepR/IRS-1/PI3K/Akt/NF-κB/p300 pathway. Similarly, knocking down IRS-1 with small interfering RNA (siRNA) or using dominant-negative mutants of p85 regulatory subunit or Akt blocked this effect. Mechanistically, leptin activates IκB kinase (IKK)α/β, leading to IκBα phosphorylation, p65 phosphorylation at Ser276, and nuclear translocation of p65 and p50, which increases κB-luciferase activity. Leptin also enhances p65/p50 binding to NF-κB sites on the IL-6 promoter, recruits the coactivator p300, and increases histone H3/H4 acetylation. This effect was blocked by antagonists against ObRb, IRS-1, PI3K/Akt, NF-κB, and p300. Altogether, these results show that leptin drives IL-6 production in microglia [138]. In primary rat microglia, leptin pre-treatment amplified cytokine release following LPS exposure, including IL-1β, TNF-α, and chemokines such as cytokine-induced neutrophil chemoattractant (CINC)-1 and MIP-2 [139].

These findings highlight leptin as a “signal 1” priming event: upon initial exposure of microglia to leptin, subsequent stimulation of TLR4 by LPS results in enhanced cytokine responses, whereas blocking JAK2 or STAT3 eliminates LPS-mediated responses [139]. Leptin also primes the nucleotide-binding oligomerization domain-like receptor family pyrin domain-containing 3 (NLRP3) inflammasome in myeloid immune cells [140]. Leptin-preconditioned microglia contain increased pro-IL-1β reserves and are hypersensitive to a secondary inflammasome stimulus, such as ATP, enabling cytokine maturation via caspase-1 [137]. Leptin also influences microglial morphology, seems to enhance microglial responsiveness to LPS by inducing morphological alterations that promote a more reactive phenotype [139]. While microglial receptors such as CD36 and complement receptors are central to phagocytosis of apoptotic debris and Aβ fibrils [141,142], direct evidence linking leptin to their upregulation is lacking. Additionally, ERK/PI3K pathways regulate ATP- and CX3CL1-mediated chemotaxis in microglia; however, whether leptin exerts modulatory effects on these migratory responses remains to be investigated [143].

Leptin can activate neuronal AMPK and Sirtuin pathways, boosting cellular metabolism [144]. It may also enhance the metabolic flexibility of microglia. Typically, resting microglia rely on oxidative phosphorylation, but when activated, they shift to glycolysis, similar to the Warburg effect, to produce energy quickly and secrete cytokines and growth factors [145]. Leptin may shift metabolism by activating mTORC1 and inhibiting AMPK, as shown in T cells [146], which enhances glucose uptake via Glut1, lactate release, and citrate production for lipid mediators [144,147]. These metabolic changes support cytokine release and ROS formation. Under conditions of energy stress, however, AMPK becomes dominant, and leptin instead promotes mitochondrial protection and cell survival, suggesting that leptin’s effects depend on the metabolic state of the cell.

Within the neurovascular unit, leptin enhances CCL production, thereby facilitating CCR2^+^ monocyte recruitment and inducing adhesion molecule expression [148]. In astrocyte-microglia co-culture, leptin-activated microglia induce astrocytic NF-κB signaling and complement release; in turn, astrocytic IL-6 enhances microglial STAT3 signaling, generating a feed-forward loop [138]. Through leptin-driven activation, microglia release pro-inflammatory cytokines such as IL-1β and TNF-α [139], which are well documented to impair hippocampal long-term potentiation and reduce dendritic spine density. Thus, leptin’s indirect influence on neurons may be mediated by these microglial cytokine pathways that disrupt synaptic plasticity [149]. Sustained exposure to leptin induces suppressors SOCS3, which prevent JAK2 phosphorylation. Overexpression of SOCS3 has been observed in microglia from obesity models, which can serve as a marker of leptin resistance. In diet-induced obesity, microglial SOCS3 is elevated, but STAT3 activation in response to leptin is blunted, while NF-κB-driven inflammation persists, demonstrating selective resistance. ER stress and receptor trafficking dysregulation further interfere with leptin signaling [150]. This combination sustains a primed inflammatory environment in which systemic leptin is elevated but unable to restore microglial damage-control function.

The ChP and BBB regulate leptin entry into the brain, so microglia, which are only exposed to parenchymal leptin, sense circulating leptin indirectly, with barrier transport kinetics determining their effective leptin signaling [151]. In obesity, hypothalamic neurons develop leptin resistance while ChP transport remains intact, chronically exposing microglia to elevated leptin [152]. In contrast, caloric restriction or GLP-1 therapies can restore LepR expression in the ChP, normalize parenchymal leptin levels, and re-establish microglial sensitivity [153].

Therapeutically, these findings indicate that leptin-microglia interactions can be targeted either by adjusting systemic leptin levels or by restoring receptor function. Systemic approaches include lowering leptin levels (via weight loss or pharmacologic modulation) to reduce SOCS3-driven resistance, or supplementing leptin in deficiency states such as cachexia [152]. Intranasal administration of leptin has shown, in a recent preclinical trial conducted with rats, higher efficiency in reaching CSF than systemic administration, potentially offering a route for circumvention of leptin transport barriers to target microglial receptors directly [154]. The “receiver strategy” aims to restore proper LepR signaling by reducing ER stress, downregulation of PTP1B, or transiently inhibiting SOCS3 to block its negative feedback on the pathway [155]. Chemical chaperones such as 4-phenylbutyrate in mouse models reduced ER stress, restored STAT3 phosphorylation in microglia, and lowered hippocampal cytokine burden [156].

Leptin exerts context-dependent effects on microglia. In chronic neurodegeneration (e.g., AD), it enhances microglial phagocytosis, reducing Aβ burden and supporting synapses [136]. In contrast, in acute neuroinflammation [e.g., relapsing remitting MS (RRMS)], leptin-primed microglia enhance antigen presentation and secrete pro-inflammatory cytokines that can activate autoreactive T cells [157]. Methodological concerns remain relevant in interpreting leptin-microglia experiments. Leptin levels are nutrition- and circadian cycle-dependent; plasma versus serum sampling varies. Most in vitro studies employ leptin at supraphysiologic concentrations, and these might potentiate different effects [158]. Obesity, which causes leptin resistance, further complicates interpretation. Collectively, leptin’s effects are context-dependent: under physiological conditions, leptin supports synaptic maintenance and phagocytosis activity, whereas in chronic metabolic or neuroinflammatory states, it drives inflammation and synaptic loss [159].

Collectively, in neutrophils, leptin primarily governs migration and survival, with activation occurring largely through indirect crosstalk and in a concentration-dependent manner. In macrophages, leptin enhance pro-inflammatory phenotype, metabolic reprogramming, and cytokine production through JAK/STAT and mTOR signaling, while also contributing to host defense under physiological conditions. Dendritic cells are particularly sensitive to leptin signaling, which promotes their maturation, survival, glycolytic reprogramming, and capacity to drive Th1/Th17-biased adaptive immune responses. In NK cells, acute leptin enhances cytotoxicity and cytokine production, whereas chronic hyperleptinemia induces functional exhaustion and impaired immunosurveillance. Within the CNS, leptin directly modulates microglial activation, inflammasome priming, metabolic remodeling, and cytokine release, with outcomes that are highly context dependent and influenced by leptin transport, receptor sensitivity, and metabolic state.

### 3.2. Leptin and Adaptive Immunity

Leptin has been shown to influence the activity of adaptive immune cells, including T lymphocytes and B lymphocytes.

#### 3.2.1. T Lymphocytes

T cells are key effectors of the adaptive immune system, acquiring diverse antigen specificity through random TCR gene recombination in the thymus. Leptin has emerged as a potent regulator of T cell activity, proliferation, and differentiation, processes critical for effective immune defense [160,161].

Leptin directly impacts thymopoiesis. Thymic cellularity is dramatically reduced in leptin-deficient *ob*/*ob* mice and leptin receptor-deficient *db*/*db* mice. This thymic atrophy is partly corrected by recombinant leptin administration, which restores thymocyte counts and normalizes double-positive (CD4^+^CD8^+^) to single-positive ratios [162]. In mice, 48 h of starvation reduces total thymocyte numbers to ~13% of fed controls, primarily through loss of cortical CD4^+^CD8^+^ thymocytes. Administration of recombinant leptin prevents this starvation-induced thymic involution. Similarly, leptin-deficient *ob/ob* mice exhibit profound thymic hypoplasia, elevated thymocyte apoptosis, and a markedly reduced CD4^+^CD8^+^/CD4^−^CD8^−^ ratio, which are all restored by peripheral leptin replacement. In contrast, weight loss alone does not rescue thymic cellularity, and in vitro leptin protects thymocytes from dexamethasone-induced apoptosis. These findings establish low circulating leptin as a key mediator of starvation-induced thymic atrophy and impaired cellular immunity [163].

In infection models, bacterial loads 72 h post-infection with *Listeria monocytogenes* were higher in *ob/ob* mice than in WT mice [164]. Additionally, during viral infection with *lymphocytic choriomeningitis virus* (LCMV), leptin-deficient mice exhibited fewer antigen-specific CD8^+^ T cells and lower IFN-γ production than WT controls [165]. The role of leptin was examined in two models of T cell-mediated hepatitis: concanavalin A (Con A) and *Pseudomonas aeruginosa* exotoxin A (PEA) administration. In both models, *ob/ob* mice were protected from liver injury and exhibited markedly reduced induction of TNF-α and IL-18 compared with WT mice. These mice also showed thymic atrophy and decreased circulating lymphocytes and monocytes. Exogenous leptin replacement restored their responsiveness to Con A and normalized lymphocyte and monocyte numbers [166].

Leptin-deficient children showed low CD4^+^ T cell counts, an inverted CD4^+^/CD8^+^ ratio, reduced naïve (CD4^+^CD45RA^+^) T cells, and impaired lymphocyte proliferation and cytokine production. Th1 cytokine IFN-γ was undetectable, while IL-4, IL-10, and TGF-β were reduced but present. Daily recombinant leptin therapy increased CD4^+^ T cells, normalized the CD4^+^/CD8^+^ ratio, raised naïve T cell numbers, and enhanced lymphocyte proliferation. It also restored IFN-γ production to normal levels, increased IL-4 and IL-10, and reduced TGF-β, correcting both T cell counts and functional responses toward a normal immune profile [161].

Leptin drives T cell subset polarization toward Th1 and Th17 phenotypes. T cell subsets rely on distinct metabolic programs: effector T cells (Teff; Th1/Th17) depend on high glucose uptake and glycolysis, whereas regulatory T cells (Tregs) rely on oxidative metabolism. Under malnutrition, a low level of circulating leptin reduced Teff number, glucose metabolism extracellular acidification rate (ECAR), and cytokine production, while leaving Treg metabolism and suppressive function unchanged. In vivo, fasting-induced hypoleptinemia in the EAE model similarly impaired Teff metabolism and function, lowering disease severity. Mechanistically, hypoxia-inducible factor (HIF)-1α, a key regulator of Th17 differentiation and glycolysis, was diminished in LepR-deficient Th17 cells and in Teff from fasted EAE mice but remained unchanged in Tregs. These findings demonstrate that leptin is cell-intrinsically required to induce glycolytic metabolism and sustain functional responses in Teff cells, but not in Tregs [167]. Human CD4^+^ T cells cultured with leptin show an increased proportion of IFN-γ- and IL-17A-producing cells, while IL-4-producing Th2 cells were decreased compared to controls [168]. Anti-leptin receptor and anti-leptin antibodies reduced the proliferation of myelin antigen-specific T cells by blocking an autocrine leptin loop that supports Th1 cell expansion, an effect dependent on leptin receptor signaling since *C57BL/Ks db/db* T cells were resistant to this inhibition [114].

In human CD4^+^ T cells, leptin increases glycolytic flux and glucose uptake relative to leptin-free controls. These metabolic shifts are accompanied by a rise in mTORC1 activity and reduced AMPK signaling, consistent with an anabolic, effector T cell phenotype [147]. Similarly, leptin-deficient murine T cells exhibit markedly reduced ECAR and impaired mTORC1 activation after antigenic stimulation, confirming leptin’s role as a metabolic licensing factor [147]. This effect is cell-intrinsic and restricted to activated effector T cells, as naïve T cells and Tregs are leptin-independent for metabolic regulation. Fasting-induced hypoleptinemia leads to persistent defects in T cell activation, with reduced glucose uptake and cytokine production even under nutrient-replete conditions. Conversely, the administration of leptin to fasted mice or the supplementation of cultured T cells from fasted donors entirely rectifies these metabolic and functional deficiencies. Moreover, forced expression of the glucose transporter Glut1 restores cytokine production in leptin-deficient T cells, underscoring that leptin promotes effector T cell function by coupling systemic nutritional status to the induction of glucose metabolism upon activation [147].

Leptin exerts a significant inhibitory effect on Tregs. In human PBMC cultures, CD4^+^CD25^+^FoxP3^+^ Tregs exposed to leptin show suppressed proliferation in response to anti-CD3/CD28 stimulation, an effect reversed by anti-leptin receptor antibodies, confirming receptor dependence [160]. In contrast, CD4^+^CD25^−^ effector T cells exhibit enhanced proliferation in response to the same leptin treatment [90,160]. In 2021, it was demonstrated that signals of pseudo-starvation reverse the anergic state of human Treg cells in vitro by activating an integrated transcriptional program that encompasses proliferation, metabolic reprogramming, and solute carrier-mediated nutrient transport. This proliferative response required induction of the cystine/glutamate antiporter solute carrier (SLC)7A11, which is transcriptionally regulated by nuclear factor erythroid 2-related factor 2 (NRF2). Notably, Treg cells from individuals with RRMS displayed impaired SLC7A11 induction, consistent with their reduced proliferative capacity. Importantly, combining in vivo dimethyl fumarate treatment with in vitro mTOR inhibition, also through leptin neutralization, reactivated NRF2, reinstated SLC7A11 expression, and rescued the proliferative potential of Treg cells from RRMS patients. These findings reveal a previously unrecognized mechanism linking leptin-driven mTOR activity to Treg cell dysfunction [169].

Follicular helper T (TFH) cells coordinate germinal center reactions that drive affinity maturation and durable humoral memory; TFH insufficiency is linked to suboptimal vaccine immunogenicity. Across healthy cohorts, lower baseline leptin associates with weaker serologic responses to influenza and hepatitis B. Leptin promotes mouse/human TFH differentiation and IL-21 production via the STAT3-mTOR axis. LepR deficiency or fasting-induced hypoleptinemia impairs TFH generation and antibody responses after immunization or infection. At the same time, leptin repletion rescues these defects, identifying low leptin as a modifiable risk factor for vaccine failure [170].

In DIO models, mice on a high-fat diet for 16 weeks reach serum leptin levels of 25–30 ng/mL, compared to 5–8 ng/mL in chow-fed controls. Tumor-infiltrating CD8^+^ T cells (TILs) from DIO mice exhibit higher PD-1 expression and secrete less IFN-γ and granzyme B per cell, as measured by intracellular flow cytometry. Functionally, these TILs are less effective in in vitro cytotoxicity against tumor targets. Notably, anti-PD-1 therapy restores cytotoxicity to within 15% of levels in controls [171,172]. These results highlight how chronic leptin elevation fosters T cell dysfunction in tumors. In other studies, using DIO mice, treatment with ObR: Fc or with anti-PD-1 increased cytokine secretion and costimulatory molecule expression on CD8^+^ T cells, improved tumor size control, and restored immunotherapy efficacy [173,174]. These results indicate that leptin-induced T cell exhaustion contributes to impaired tumor control in obesity and aging but is pharmacologically reversible.

In non-obese diabetic (NOD) mice, autoimmune insulitis is associated with elevated leptin levels. Leptin-treated female NOD mice exhibited accelerated type 1 diabetes onset and mortality, along with enhanced Th1 responses, evidenced by abundant IFN-γ mRNA-expressing cells in splenic periarteriolar sheaths despite no significant change in overall IFN-γ or IL-4 secretion from stimulated splenic T cells compared to controls [175]. These findings indicate that leptin neutralization can rebalance T cell function and delay autoimmunity. In a collagen-induced arthritis (CIA) model, leptin treatment increased joint swelling and raised serum autoantibody titers by 1.8-fold at week 8 post-immunization. These effects were accompanied by enhanced Th17 and Th1 polarization in CD4^+^ cells (IL-17^+^IFN-γ^+^ double-high populations), demonstrating leptin’s ability to amplify autoimmune T cell responses [176]. Similarly, both *ob*/*ob* and *db*/*db* mice developed markedly less severe antigen-induced arthritis, with reduced synovial IL-1β and TNF-α expression. They show diminished antigen-specific T cell proliferation, lower IFN-γ, higher IL-10, and reduced anti-methylated BSA antibody levels [177].

Collectively, leptin replacement in deficiency states can restore T cell survival, metabolism, and memory, thereby enhancing vaccine responses and resistance to infection. Conversely, leptin blockade or LepR antagonism in obesity or autoimmunity can rebalance the activity of effector/regulatory T cells, reduce pathogenic cytokine production, and enhance immune regulation. Targeting downstream effector pathways (e.g., JAK2/STAT3, mTORC1) or using leptin sensitizers (e.g., SOCS3 inhibition, ER stress relief) offers additional strategies to selectively modulate T cell responses independently of systemic leptin deregulation.

#### 3.2.2. B Lymphocytes

B cells are central components of the adaptive immune response; beyond producing antibodies, they also present antigens, secrete cytokines, and regulate immune activity [178]. Leptin can affect B cells’ survival, differentiation, and function through leptin receptor, ObRb, expressed on both human and murine B cells [179].

Leptin activates JAK2 and STAT3 phosphorylation, followed by ERK1/2, p38 MAPK, and PI3K/Akt activation in in vitro studies of human peripheral B cells [179]. Functionally, this signaling cascade induces a pro-inflammatory transcriptional response, including increased secretion of IL-6, TNF-α, and IL-10, along with upregulation of surface antigens such as CD25 and HLA-DR in a concentration-dependent manner. All these functions are abrogated when JAK2, STAT3, or MAPK pathways are pharmacologically inhibited [179]. Leptin also stimulated aged human B cells to produce higher levels of IL-6, TNF-α, and IL-10 than young controls, accompanied by enhanced STAT3 phosphorylation, suggesting that leptin-driven B cell cytokine production may contribute to age-related chronic inflammation [180].

Leptin’s role in B cell growth and survival has also been demonstrated in vivo. Leptin-deficient *ob*/*ob* mice and leptin receptor-deficient *db*/*db* mice show reduced numbers of B cells in the spleen and bone marrow, increased proportions of immature transitional B cells, and higher rates of apoptosis [181,182]. An experiment in 2021 demonstrated that B-lymphocyte numbers were reduced by approximately 70% in leptin-deficient *ob*/*ob* mice compared with WT controls, an effect restored by leptin replacement therapy [182]. Exogenous leptin treatment also revives B-cell cellularity. It induces anti-apoptotic protein expression (e.g., Bcl-2) and cell-cycle regulators (e.g., Cyclin-D1), which would be expected from survival pathways induced by leptin in B cells [181].

Based on leptin’s role as an immune activator, it has been proposed that low physiological levels of leptin can modestly enhance B cell function, including activation-induced cytidine deaminase (AID) expression and class-switch recombination (CSR), in specific contexts. However, chronic exposure to elevated leptin, as seen in obesity or aging, has been shown to suppress CSR-related molecules. Consistent with this, young and elderly individuals with obesity exhibited reduced influenza vaccine responses, with fewer switched memory and transitional B cells and an accumulation of pro-inflammatory exhausted memory B cells. B cells from these individuals showed impaired antibody response capacity, with lower AID and E47 expression, increased IL-6 and decreased IL-10 secretion, and elevated basal activation markers such as TNF-α, TLR4, and microRNAs, which negatively correlated with B cell function [183]. Exposing B cells from lean individuals to leptin reproduced this dysfunctional phenotype by increasing phospho-STAT3 (driving TNF-α production) and reducing phospho-AMPK (an upstream regulator of p38 MAPK and E47), indicating that high leptin contributes to obesity-associated B cell inflammation and impaired vaccine responses [183]. In B cells from young lean adults, leptin expands pro-inflammatory B-cell subsets and induces intrinsic inflammation (TNF-α, IL-6, IL-8; miR-155/miR-16; TLR4; CDKN2A/p16INK4a). Baseline expression of these markers correlates negatively with subsequent B-cell responses to in vivo/in vitro stimulation. Leptin suppresses class-switch recombination and reduces influenza vaccine-specific IgG production. Thus, leptin drives a phenotype in young lean B cells resembling that of young obese and elderly individuals, implicating leptin as a mediator of B-cell immunosenescence [184].

In autoimmune disease, leptin’s pro-inflammatory effects likely promote the persistence of autoreactive B cells [185]. While direct in vivo models of leptin-mediated autoimmune antibody production are limited, clinical evidence shows elevated leptin levels in human autoimmune diseases such as rheumatoid arthritis (RA), systemic lupus erythematosus (SLE) and MS [186]. In RA, synovial fluid contains higher leptin concentrations than plasma, and local antigen-presenting cells (e.g., DC-like subsets) demonstrate enhanced IL-12 production, reflecting leptin’s role in shaping autoreactive B cell-preferential inflammatory microenvironments [185].

These findings suggest that leptin is an immune-metabolic regulator of B cell biology. Leptin promotes survival, drives inflammatory effector functions, reprograms metabolism toward glycolysis, and controls antibody responses, thereby shifting immunity away from germinal center-dependent humoral responses toward extrafollicular, cytokine-driven events during chronic hyperleptinemia [187]. Physiologically supportive of competent immunity, high leptin level in excessive or chronic states has the potential to destabilize antibody quality and promote autoimmunity [188]. Therapeutic interventions aimed at restoring balance by suppressing leptin in chronic inflammation, replenishing it when deficient, or normalizing downstream signaling (JAK-STAT, mTORC1) may enhance vaccine responsiveness, reverse obesity-associated immunocompromise, and restore B cell tolerance in autoimmunity.

Collectively, in T cells, leptin sustains thymic cellularity, supports effector T cell survival, and licenses Teff (Th1/Th17 and CD8^+^) metabolism through mTORC1-driven glycolysis, while simultaneously constraining Treg proliferation and function. Physiologically, this coupling of systemic nutritional status to T cell metabolism preserves host defense, enhances vaccine responsiveness, and supports formation of TFH and memory compartments. However, in chronic hyperleptinemic states such as obesity and some autoimmune settings, the same pathways promote T cell exhaustion, and loss of regulatory control, thereby amplifying tissue inflammation, and impairing anti-tumor immunity. In B cells, leptin similarly promote survival, pro-inflammatory cytokine production, and metabolic activation via JAK2/STAT3, MAPK, and PI3K/Akt signaling. While physiological leptin levels support B cell homeostasis and may modestly enhance functional responses, chronic elevation drives an intrinsically inflamed, metabolically reprogrammed B cell phenotype characterized by reduced class-switch recombination, impaired vaccine responses, and expansion of exhausted, pro-inflammatory subsets.

## 4. Leptin and EAE

EAE is a widely used mouse model for studying autoimmune neuroinflammatory disease of the CNS. Specifically, it allows for the infiltration of T lymphocytes and myeloid cells into the CNS, creating lesions characteristic of demyelination and inflammation, which is consistent with diseases such as MS [189].

The course of EAE is determined by the genetic background and the specific myelin antigen used for induction. We employ the myelin glycoprotein (MOG_35–55_) model in *C57BL*/*6* mice for studying chronic MS, while using proteolipid protein (PLP_139–151_, PLP_178–191_) in *SJL*/*J* mice mimics RRMS [190]. In this active EAE model, pertussis toxin is administered alongside the myelin antigen to prime myelin-specific CD4^+^ T cells, which then migrate to the CNS and initiate autoimmune inflammation [189]. In contrast, passive induction involves transferring in vitro-stimulated lymphocytes from immunized mice into naïve mice, resulting in a faster and more severe disease onset [190].

The role of leptin in EAE has been explored through studies examining its secretion patterns and functional impact on disease progression.

### 4.1. Leptin Dynamics in EAE and WT Controls

A 2003 study evaluated the kinetics of leptin level in EAE in WT controls in both *C57BL*/*6J* and *SJL*/*J* mice after immunization. The study found a significant surge in leptin levels before the onset of the acute phase of EAE. The leptin level rise started after immunization but only persisted until peak disease in *SJL*/*J* females, with significantly lower levels in male *SJL*/*J* mice. The increase in serum leptin was associated with greater disease susceptibility, reduced food intake, and loss of body weight [114]. Another study on EAE showed that serum leptin levels in *C57BL*/*6* mice lost the normal circadian rhythm seen in controls, emphasizing the need to measure leptin at consistent times to account for circadian effects and suggesting that disrupted leptin signaling may contribute to metabolic issues, sleep disturbance, and fatigue in MS [191].

The influence of leptin and sex hormones on EAE susceptibility was explored in a 2001 study using *SJL*/*J* mice. Male mice were divided into three groups: receiving PBS (days 0–10), recombinant leptin given before and after priming, or recombinant leptin given continuously (days 0–10). Leptin treatment increased EAE incidence, severity, and mortality, with cellular infiltration in the brain and spinal cord. In females, leptin also worsened disease, as shown by higher clinical scores, greater weight loss, more CNS infiltration, and higher mortality. In males, leptin given after priming did not significantly change disease resistance, whereas in females, post-priming, disease severity rose but less strongly than during immunization. Leptin also enhanced delayed-type hypersensitivity (DTH): in males, responses reached the level of PBS-treated females, while in females, leptin caused a substantial increase compared with controls. Cytokine analysis revealed that in leptin-treated males, secretion shifted from a Th2 to a Th1 pattern, while in females, leptin markedly increased IFN-γ with little change in IL-4. These findings suggest that leptin, in conjunction with sex hormones, contributes to sex-specific differences in EAE susceptibility [192].

Further examining leptin concentration patterns, a 2013 study examined leptin concentration patterns and demonstrated increased leptin receptor expression in reactive astrocytes of the hippocampus in EAE mice. To test whether EAE induces leptin upregulation across the BBB, *SJL*/*J* female mice with EAE were evaluated using two approaches: (1) intravenous injection of radiolabeled leptin (^125I-leptin) and albumin (^131I-albumin), and (2) in situ brain perfusion with a tracer to measure transport directly. Unlabeled leptin was co-administered as a competitive inhibitor to confirm specificity. As expected, ^131I-albumin uptake increased due to BBB permeability, but importantly, leptin transport across the BBB was persistent and saturable. In the intravenous (IV) experiments, leptin uptake was significantly elevated in the hippocampus and cervical spinal cord 21 days post-immunization, and this effect was entirely blocked by excess unlabeled leptin, confirming receptor mediation. These findings show sustained, receptor-mediated leptin transport across the BBB in EAE, highlighting leptin’s role in neuroinflammatory signaling [193].

In 2012, a study examined changes in astrocytic LepR expression across brain regions in *SJL*/*J* mice with EAE. Immunohistochemistry revealed markedly higher LepR in astrocytes of the hippocampus, dorsomedial hypothalamus, and arcuate nucleus. Protein analyses showed increased GFAP and LepR expression in hippocampal tissue 16 days after EAE induction. In hippocampal homogenates, ObRa mRNA was elevated at day 24 (resolution phase), whereas ObRb mRNA significantly decreased at day 12 (peak disease). These results suggest that astrocytic LepR expression increases notably in the hypothalamus and hippocampus, potentially shifting leptin signaling toward a neuroinflammatory role [194].

### 4.2. Leptin and Leptin-Receptor Knockout EAE Experiments

Beyond studies examining leptin levels in EAE, some evaluated disease outcomes in leptin- or leptin receptor-knockout mice. A 2001 study tested whether leptin is required for the induction and maintenance of EAE. Experimental groups consisted of *ob*/*ob* mice without leptin replacement, *ob*/*ob* mice with leptin replacement, and WT controls. After EAE induction with MOG, leptin-deficient mice without leptin replacement were resistant to disease, whereas leptin-restored *ob*/*ob* mice became susceptible, similar to WT mice. Additionally, there was a shift from a Th2 to a Th1 cytokine profile, characterized by increased IFN-γ production. These findings demonstrate that leptin is essential for promoting the Th1-mediated proinflammatory responses characteristic of EAE [195]. Similarly, another study reported that, unlike WT mice which developed progressive paralysis following MOG immunization or adoptive transfer of encephalitogenic cells, leptin-deficient *C57BL*/*6J ob*/*ob* mice were resistant to EAE induction [114]. This protection was associated with impaired maintenance of MOG-specific CD4^+^ T cells, indicating a critical role for leptin in sustaining autoreactive T-cell survival, accompanied by marked downregulation of the survival factor Bcl-2. Administration of exogenous leptin restored both T-cell survival and disease susceptibility. Furthermore, treatment of WT mice with ObR: Fc increased the proportion of Tregs and attenuated both the clinical severity and progression of disease in *SJL/J* EAE mice [157].

A 2015 study investigated how endothelial leptin signaling influences the course of EAE using endothelial leptin receptor knockout (ELKO) mice. Female *C57BL*/*6* mice carrying the ELKO mutation were compared with WT littermate controls. During EAE, there was an early upregulation of LepR isoforms before disease onset, followed by inhibition of gene expression that persisted to day 24 in spinal cord samples. Clinically, ELKO mice displayed lower scores on days 12–20, and their cumulative scores over the peak 10 days of disease were also significantly reduced, indicating decreased disease severity. WT-EAE mice showed higher sodium fluorescein leakage in both the spinal cord and the brain compared with ELKO-EAE mice. Moreover, at later disease stages, WT-EAE mice showed higher percentages of CD4^+^ T cells, CD8^+^ T cells, and CD11b^+^Gr1^+^ granulocytes compared with ELKO-EAE mice. Altogether, ELKO-EAE mice exhibited reduced BSCB permeability and inflammatory cell infiltration, suggesting that endothelial leptin signaling promotes EAE pathogenesis [94].

To investigate the consequences of astrocytic leptin signaling, researchers in 2013 analyzed EAE progression in astrocyte-specific LepR knockout (ALKO) mice compared with WT controls. Disruption of astrocytic leptin signaling did not affect EAE onset or early-stage progression but significantly impaired recovery. ALKO mice exhibited higher clinical scores, greater cell infiltration, increased demyelination, and enhanced T cell activation. These results point to a neuroprotective role for astrocytic leptin signaling during EAE recovery [196].

### 4.3. Therapeutic Modulation of Leptin in EAE

A 2006 study tested whether blocking leptin could reduce EAE severity using anti-leptin antibodies or ObR: Fc. Female *SJL*/*J* mice were induced with EAE and treated with PBS or control Ig, or with anti-leptin antibodies or ObR: Fc either at the time of immunization or at disease onset. An adoptive transfer model was also included. Leptin blockade significantly reduced disease severity, delayed onset, slowed progression, and decreased relapses. DTH responses were inhibited, and lymph node cultures with PLP showed reduced T cell proliferation in treated mice. Cytokine profiles shifted toward a Th2 phenotype, with increased Foxp3 expression in CD4^+^ T cells, supporting enhanced Treg development. Ex vivo, CD4^+^ T cells from ObR: Fc-treated mice failed to downregulate p27, correlating with increased ERK1/2 phosphorylation, and displayed elevated STAT6 phosphorylation, consistent with Th2 cytokine switching. Collectively, these findings demonstrate that leptin blockade ameliorates EAE by reducing Th1 responses and promoting Th2/Treg pathways [197].

Following early evidences implicating leptin in EAE susceptibility, subsequent research examined the effects of fasting and calorie restriction. A 2008 study investigated the impact of diet on hormone levels and EAE progression in *SJL*/*J* and *C57BL*/*6* mice fed either a normal, 40% calorie-restricted, or high-fat/high-calorie diet. Calorie-restricted mice developed significantly milder disease, with *SJL*/*J* survival reaching 80% compared to 30% in the high-fat/high-calorie group and 23% under regular feeding. Histology confirmed reduced inflammation, demyelination, and axonal injury in calorie-restricted animals. Calorie restriction lowered plasma leptin while increasing corticosterone and adiponectin and decreasing IL-6 [198].

In a 2016 study, *C57BL*/*6J* mice were fed either normally or fasted for two days with or without twice-daily leptin injections. EAE was then induced to assess the effects of leptin. It was found that fasting-induced hypoleptinemia reduced the number of Teff cells, proliferation capacity, and glucose metabolism. Notably, the numbers and function of Treg cells remained unaffected by fasting, highlighting the leptin requirement for Teff cells but not for Treg cells. Furthermore, hypoleptinemia reduced the expression of hypoxia-inducible factor (HIF)-1α, a protein associated with inflammatory processes in EAE, in Teff cells. Overall, there was a notable decrease in disease severity in EAE in the fasted mice without leptin treatment associated with suppressed Teff cell function without the simultaneous suppression of Treg cells, hinting at the idea that leptin supports the pathogenic Teff cell processes which drive EAE pathology [167].

Taken together, EAE studies place leptin as a pro-inflammatory, disease-amplifying hormone, providing a strong rationale to examine whether similar leptin-driven immune programs operate in MS.

## 5. Leptin and MS

**Figure 2:**
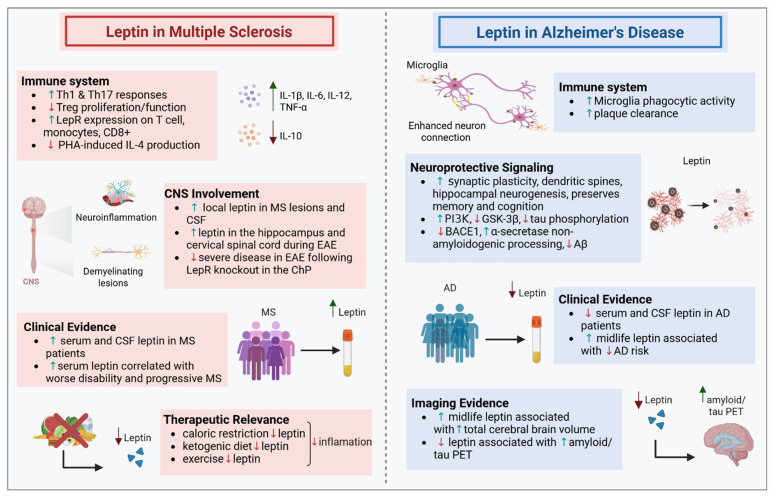
**Role of leptin in the pathogenesis of Multiple Sclerosis and Alzheimer’s disease.** (**Left panel**): In MS, high leptin level enhances Th1/Th17 responses, suppresses Tregs, and drives CNS inflammation, with high serum levels correlating with relapses, disability, and progression. (**Right panel**): In AD, leptin activates neuroprotective pathways, reduces Aβ and tau pathology, and supports synaptic plasticity; higher midlife leptin levels are associated with a lower AD risk (Schematic created with BioRender).

MS is a chronic autoimmune disease of the CNS characterized by inflammation, demyelination, axonal damage, and neurodegeneration. MS involves immune cell infiltration, including T cells, B cells, and myeloid cells, into the CNS, inducing local lesions, tissue injury, and inflammation [199]. Inflammatory pathways have a central position in the pathogenesis of MS, and growing evidence indicates that metabolic diseases such as obesity significantly affect immune function and possibly risk and progression of MS [200] (Figure 2).

### 5.1. Leptin as a Link Between Metabolism and Immune Response in MS

As stated above, leptin is a key mediator linking metabolic and inflammatory processes, exerting pleiotropic immunomodulatory effects. Overall, higher leptin levels promote a pro-inflammatory immune phenotype by enhancing Th1 and Th17 activation and proliferation while inhibiting Treg expansion [160]. The immune-metabolic role of leptin has generated considerable interest in its therapeutic potential for influencing the course and pathogenesis of MS. Leptin has a significant impact on cytokine release and immune polarization.

In experimental evidence, recombinant leptin in PBMCs from RRMS patients stimulated an increase in TNF-α, IFN-γ, IL-6, and IL-10 secretion, particularly in those with relapses. In contrast, this response was absent or minimal in stable MS patients and healthy controls [6,201]. In another experiment, the addition of recombinant leptin to PBMC cultures from MS patients in vitro induced a dose-dependent increase in IL-10 secretion and a dose-independent reduction in IFN-γ production. TNF-α levels decreased at the lowest recombinant leptin concentration, but this effect was reversed at higher doses. Recombinant leptin did not stimulate IL-2 or IL-4 production at any concentration tested [202]. Interestingly, monocytes from RRMS patients were leptin-nonresponsive, showing no significant cytokine induction, whereas PBMCs remained responsive, suggesting cell-specific and disease stage-dependent leptin sensitivity [6]. Additionally, leptin suppressed phytohemagglutinin (PHA)-induced IL-4 production by PBMCs of patients in an acute phase of disease and anti-inflammatory signaling [201].

Supporting the role of leptin in MS, transcriptomic studies revealed upregulation of ObRb on activated human T cells and positive feedback between leptin signaling and effector T cell activation [147]. The ObRb expression is shown to be enhanced on CD8^+^ T cells and monocytes during active disease phases of MS, which implies that leptin signaling is part of disease activity and immune dysregulation [9]. Collectively, these findings support leptin’s role in shifting immune responses toward Th1/Th17 polarization, a well-established pathogenic pathway in MS.

Inhibition of Treg cells represents one of the primary mechanisms through which leptin exacerbates MS. Different studies showed an inverse correlation of high circulating leptin levels with CD4^+^CD25^+^FoxP3^+^ Treg frequency in both MS patients and controls [157,203,204]. Leptin signaling was blocked experimentally using intraperitoneal neutralizing antibodies, which restored Treg proliferation and reduced autoreactive CD4^+^ T-cell responses to myelin antigens in vitro [157].

Leptin has also been discovered to be in higher concentration in post-mortem CNS MS lesions, suggesting localized involvement in the neuroinflammatory microenvironment [205]. In RRMS patients who were naive to therapy, one study measured leptin levels in both the serum and CSF and found a substantial increase in both in comparison to controls. Leptin levels in the serum of RRMS patients were positively correlated with CSF IFN-γ levels, indicating a pro-inflammatory immune phenotype [9,157]. This effect was confirmed by a functional assay showing that neutralizing antibodies against leptin or its receptor reduced the proliferation of autoreactive T cell lines stimulated with human myelin basic protein. The study also demonstrated that LepR expression increased on T cells following activation, supporting leptin’s role as a secondary signal in autoimmune T cell activation [160].

**Figure 3:**
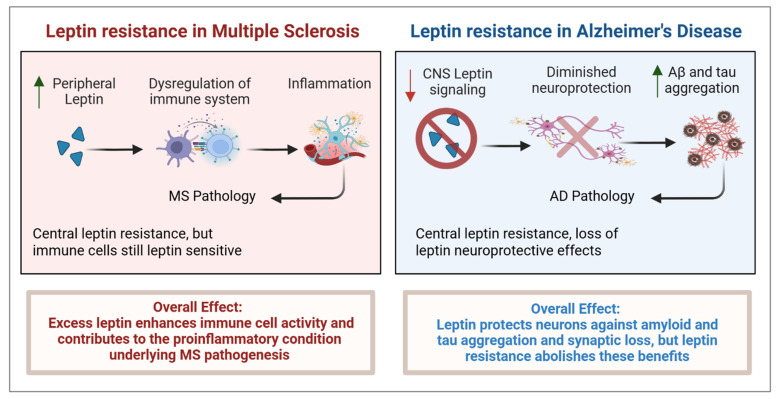
**Role of leptin resistance in the pathogenesis of Multiple Sclerosis and Alzheimer’s disease.** Leptin resistance sustains peripheral inflammation and limits CNS leptin signaling, leading to enhanced autoimmunity in MS and reduced neuroprotection in AD (Schematic created with BioRender).

In leptin-resistant conditions, central leptin signaling and CNS responsiveness are reduced, whereas peripheral leukocyte responses to leptin remain intact [206]. However, some discrepancies have been reported. For instance, NK cells may also develop leptin resistance under certain conditions [126,127]. It has been reported that in RRMS patients with average BMI, SOCS3 expression is reduced in leptin-sensitive immune cells during relapses, permitting unchecked leptin-induced inflammatory signaling [9] (Figure 3).

### 5.2. Leptin Levels Across MS Phenotypes

Most studies report elevated leptin levels across different MS subtypes, with patients consistently showing significantly higher concentrations than healthy controls [9,74,203,204,207,208,209,210].

In a case–control study, 191 MS patients and 200 age- and sex-matched healthy controls were recruited, and serum leptin levels were found to be significantly higher in the MS group compared to controls [204]. Similarly, in 2024, researchers evaluated serum leptin levels in RRMS patients and controls using Enzyme-Linked Immunosorbent Assay (ELISA) [209]. They reported a mean level of 42,441.5 ± 571.5 pg/mL in RRMS patients compared to 32,461.03 ± 446.36 pg/mL in controls, confirming a significant elevation in circulating leptin in RRMS patients [209]. A study involving 25 patients with RRMS and 25 age- and sex-matched healthy controls investigated the relationship between leptin and Tregs. Blood samples were collected within two weeks of acute relapse onset in the RRMS group. BMI did not differ significantly between patients and controls, irrespective of sex. However, patients with RRMS exhibited markedly higher serum leptin levels than controls, accompanied by significantly reduced Treg cell frequencies and lower mean fluorescence channel values of FoxP3 expression [203]. Additionally, upregulation of ObRb on CD8^+^ T cells and monocytes in RRMS patients during relapse, relative to remission and control groups, further supports the involvement of leptin signaling in mediating relapse activity [9].

In 2013, a cross-sectional study examined leptin levels across three subtypes of adult-onset MS, RRMS, primary-progressive MS (PPMS), and secondary-progressive MS (SPMS), and compared them with healthy controls. After adjusting for age and batch effects, leptin levels differed significantly among all groups and healthy controls, with the highest levels observed in SPMS. This difference was attenuated after additional adjustment for BMI, though leptin remained strongly associated with BMI across all participants [74]. These findings reinforce the association between higher leptin and progressive disease phenotypes. However, this study found no significant differences in leptin levels between patients in the relapse and remission phases [74]. Also, another study in 2021 reported a higher level of leptin in SPMS than other subtypes of MS [210].

Findings on leptin levels in MS have been variable, as some findings were in contradiction depending on the study population, disease stage, and method of analysis. A 2021 Turkish clinical study that used a cross-sectional study design found significantly lower serum leptin in MS patients (6.12 ± 5.34 ng/mL) than in controls (13.02 ± 8.25 ng/mL), even after excluding individuals with diabetes, dyslipidemia, or liver dysfunction [211]. Similarly, other studies found no significant difference in serum leptin levels between newly diagnosed RRMS patients or CIS patients and healthy controls [202,212,213,214]. Additionally, Mendelian randomization (MR) analysis was used in a study to estimate the effect of leptin levels on MS risk in a large cohort. Genetic susceptibility to increased BMI correlates with a greater risk of MS, but no causal effect was detected for circulating leptin levels [215].

These inconsistencies highlight the need for standardized assays, cohort matching, and stratification by sex, age, BMI, treatment status, and disease duration or type. Notably, a recent meta-analysis of 645 MS patients and 586 controls confirmed a significant elevation of leptin levels in MS (SMD = 0.70; 95% CI = 0.24–1.15) [216].

Leptin has also been assessed as a prognostic factor in MS in relation to treatment response. A study in 2005 evaluating SPMS patients undergoing IFN-β therapy employed a prospective longitudinal design to monitor both biochemical and clinical outcomes over a 12-month treatment period. Serum leptin was quantified via ELISA at baseline and follow-up. At the same time, disability status was assessed at the same visits using the Expanded Disability Status Scale (EDSS) and corroborated timed 25-foot walk and Nine-Hole Peg Test performance. Among patients whose EDSS scores remained stable throughout the year, indicating no confirmed disability progression, leptin levels decreased significantly, paralleled by reductions in circulating IL-6. In contrast, patients classified as progressive (defined by a sustained EDSS increase of ≥1.0 point confirmed at 6 months) exhibited no significant changes in leptin or IL-6, despite identical treatment exposure and BMI stability [217]. This suggests that a decline in leptin levels may reflect a favorable immune-metabolic response linked to clinical stability. In contrast, persistently elevated leptin could be associated with continued inflammatory activity or treatment non-responsiveness. In a RRMS longitudinal trial, relapse-free IFN-β-treated patients showed dramatic decreases in serum leptin, while relapsing patients had pre-exacerbation leptin increases [208]. However, an observational cohort study measuring serum leptin before and after dimethyl fumarate therapy found that serum leptin levels did not correlate with the efficacy of therapy [218].

Serum leptin levels have been shown to correlate positively with EDSS scores and disease progression [210]. Another study demonstrated this association in PPMS and RRMS phenotypes but not in SPMS [219]. It was also shown that leptin levels were raised in clinically disabled RRMS patients [204], and the correlation held even after controlling for body composition. Similarly, regarding leptin’s prognostic value, one study found that in male MS patients, higher serum leptin was associated with more severe optic neuritis, but not with recovery [220]. Leptin levels were shown to be particularly predictive of disability when paired with other inflammatory adipokines, such as resistin [204,219]. Nonetheless, some studies failed to find any such correlation between leptin and EDSS or magnetic resonance imaging (MRI) disease activity. That correlation between clinical outcomes and leptin is variable depending on the stage of the disease or treatment [221,222,223,224]. This may suggest that leptin’s association with clinical measures may be stronger in progressive or untreated patients and further emphasizes heterogeneity in patient cohorts and disease-modifying therapy exposure [221,222]. A study in 2013 showed that the leptin-BMI relationship is disrupted in MS patients with higher EDSS scores, indicating the possibility of leptin production unrelated to adiposity due to inflammation [225].

### 5.3. Age, Sex, and Genetics in the Context of Leptin-Mediated Risk in MS

Sex-specificity of leptin expression has been assessed in different studies. In most of the studies, baseline serum leptin levels were greater in females than in males among normal controls and MS patients [213,223]. Pediatric literature for MS also shows sex-differentiated divergent function: higher leptin in boys was associated with a longer relapse-free interval, while higher leptin in girls was associated with higher levels of disability/increased EDSS scores [90,226,227].

A 2020 study using prospectively collected samples from a Swedish biobank, pre-symptomatic serum samples from 649 individuals who later developed MS were compared with 649 matched controls. In individuals under 20 years of age, higher leptin concentration was associated with higher MS risk within the <20 years age group (OR  =  1.4, 95% CI  =  1.1–1.9) and all men. This was contrasted with decreased risk of MS in females aged 30–39 years with higher leptin levels, demonstrating sex- and age-dependent effects of leptin. These findings suggest a particularly susceptible window period in adolescence, independent of sex and age, in the association between leptin and MS risk [227].

Leptin and LepR gene polymorphisms also confirm leptin’s role in MS. Data indicated that, in a sex-dependent manner, the single nucleotide polymorphisms (SNP) in *LEP*, rs2167270 G allele, which produce less leptin was associated with reduced susceptibility to MS. Additionally, high leptin producer rs7799039AA genotype was found to be significantly more frequent in male MS patients than in male controls [228]. Additionally, in a Kuwaiti cohort, the *LEP* rs7799039AA genotype was significantly associated with a higher risk of MS (OR = 2.52; 95% CI = 1.35–4.67). However, there was no influence of the rs7799039AA genotype on plasma leptin level. Moreover, in this study, MS patients had significantly lower plasma leptin levels than controls, suggesting that future studies should account for all factors influencing leptin levels to clarify its controversial outcomes in MS pathogenesis and progression [229].

In 2021, analysis of genotype distributions for the three SNPs (*LEP* rs7799039, *LEPR* rs1137101, and *PGC1A* rs8192678) in patients with RRMS or SPMS and controls revealed that only the *PGC1A* rs8192678 minor allele was linked to a higher MS risk [230].

A study examining the *LEP* G-2548-A and *LEPR* 223A/G polymorphisms reported significant associations between *LEP* and *LEPR* polymorphisms and MS susceptibility. *LEP* and *LEPR* variants emerged as key predictors of elevated circulating leptin in MS patients, with the GG genotype correlating with higher leptin concentrations [231].

Genetic and transcriptomic studies have shown that leptin and resistin are regulators in inflammatory networks associated with MS [204,229,230]. While Mendelian randomization analysis suggests that the role of leptin in MS disease is non-causal, pleotropic or modulatory relations are possible [232].

### 5.4. Therapeutic Implications of Leptin in MS and Future Directions

Given this heterogeneity, the overall evidence favors a model where leptin is a potent immune-metabolic signal to drive MS risk, relapse activity, disability progression, and treatment response [233]. While still not an established biomarker or target for therapy, leptin is a potential candidate for personalized medicine therapies, especially in metabolically susceptible subgroups or during an immune susceptibility window such as adolescence or after pregnancy [234].

Metabolic interventions modulate leptin dynamics. Calorie restriction reduced serum leptin and increased circulating CD45RO^+^ Tregs within 6 weeks, paralleled by improvement in cognitive function on the paced auditory serial addition test (PASAT) [235]. Ketogenic diet reduced serum leptin by 35% and decreased IL-17 levels, with the effect appearing within three months of therapeutic diet [236]. These changes were accompanied by significant improvements in EDSS score, fatigue, 6 min walk test, and Nine-Hole Peg Test performance [236]. Exercise training, likewise, had the identical reducing effect on leptin and proinflammatory cytokines, including TNF-α and IL-6, suggesting a reversible regulation mechanism of the disease [237,238].

Recent meta-analysis of current clinical studies in obesity demonstrates that GLP-1RA attenuates weight loss and decreases leptin serum levels [239]. This effect may suggest that GLP-1RAs could indirectly modulate leptin sensitivity in other disease contexts, such as neuroinflammation. In MS and EAE, GLP-1RAs have shown promise in both reducing disease risk and improving clinical symptoms [240,241,242,243,244,245]. Although the exact mechanisms remain unclear, one possibility is that by lowering hyperleptinemia, GLP-1RAs attenuate T cell activation and thereby limit pathogenic Th17 responses [246]. Future research will need to associate leptin signaling with metabolic and immunologic data to map its extension on MS pathobiology.

## 6. Leptin and AD

AD is a chronic progressive neurodegenerative disorder and the leading cause of dementia worldwide [247,248]. The hallmark pathological features of AD include extracellular deposition of Aβ aggregates in neuritic plaques (amyloid plaques), intracellular accumulation of hyperphosphorylated and aggregated tau in neurofibrillary tangles (NFTs), neuronal and synaptic loss, and widespread neuroinflammation [249].

AD is associated with an active immune response within the CNS, primarily mediated by microglia and astrocytes. Microglia detect amyloid deposits through pattern recognition receptors, including TREM2 and TLR, and subsequently adopt pro-inflammatory phenotypes, releasing cytokines (e.g., IL-1β, TNF-α, IL-6) and ROS. While these responses aim to facilitate aggregate clearance, persistent microglial activation can worsen synaptic dysfunction, accelerate amyloid and tau pathology, and impair neuronal repair. Furthermore, monocytes, T lymphocytes, and other peripheral immune cells may infiltrate the brain through a disrupted BBB, thereby amplifying neuroinflammation and altering disease progression [250].

Although amyloid and tau remain the dominant pathogenic models in AD, increasing evidence highlights the influence of systemic metabolic factors, such as adipokine signaling, including leptin, on brain vulnerability decades before clinical onset [251] (Figure 2).

### 6.1. Protective Role of Leptin in AD

Leptin signaling is essential for normal brain development and memory function. Its loss in leptin- and leptin receptor-deficient mice leads to reduced brain volume, impaired neurogenesis, and memory deficits. Remarkably, restoring leptin receptor expression in adulthood reverses these abnormalities, including brain atrophy and cognitive impairment [252]. Leptin has recently been recognized as a pleiotropic neuromodulator with direct effects on hippocampal and cortical neurons [253]. Through ObRb receptor in various brain regions, leptin activates JAK2/STAT3, PI3K/Akt, and AMPK signaling pathways, while inhibiting glycogen synthase kinase-3β (GSK3β) [254]. These signaling pathways intersect with the molecular processes driving AD pathology: JAK2/STAT3 and PI3K/Akt activation promote anti-apoptotic and pro-survival gene transcription, while AMPK activation and GSK-3β inhibition reduce tau hyperphosphorylation, stabilize microtubules, prevent NFT formation, and decreased soluble Aβ [255,256].

Experimental evidence shows that leptin downregulates the expression of Beta-site amyloid precursor protein cleaving enzyme 1 (BACE1), increases the processing of APP via non-amyloidogenic pathways, and reduces the production of Aβ1-40 and Aβ1-42 [257]. Importantly, BACE1 inhibition in mice has been shown to restore leptin sensitivity, normalize hypothalamic inflammation, and reduce the expression of PTP1B and SOCS3 [258]. However, these negative regulators, such as SOCS3 and PTP1B, which disrupt JAK2/ObRb signaling, are upregulated in the in APP/PS1 hippocampus [259], contributing to central leptin resistance despite preserved peripheral levels. The APP/PS1 double-transgenic mice, carrying human APP (Swedish mutation) and PS1 (exon 9 deletion), are a widely used AD model that develops early amyloid plaque deposition, along with gliosis, synaptic dysfunction, and progressive cognitive deficits [260].

Leptin also enhances the phagocytic capacity of microglia, enabling more efficient clearance of extracellular amyloid plaques, while simultaneously attenuating microglial secretion of pro-inflammatory cytokines such as IL-1β and TNF-α that can exacerbate synaptic dysfunction [261]. In human neuronal cultures, experimental evidence shows that leptin reduces extracellular Aβ accumulation and prevents synaptic loss caused by oligomers. Likewise, in transgenic mouse models, leptin administration reduces Aβ burden in the cortex and hippocampus, while also enhancing long-term potentiation and memory performance [262]. Collectively, leptin influences multiple pathological processes in AD, including the regulation of amyloid production and clearance, attenuation of tau pathology, inhibition of maladaptive neuroinflammation, and maintenance of synaptic network integrity, underscoring its potential as a therapeutic target.

### 6.2. Leptin Signaling Dysfunction in AD and Experimental Animal Models of AD

Body weight and metabolic status are essential modifiers of AD risk. Midlife obesity has been associated with a greater risk of dementia and cognitive decline [254,263,264]. In contrast, weight loss in late life may serve as an early manifestation of AD-related brain dysfunction [254,263,264]. Low BMI has been linked to worsening AD pathology and abnormal CSF biomarkers, including tau and Aβ1-42 [265]. Several studies report that low plasma leptin levels in older adults, or in patients with AD, are associated with cognitive decline, abnormal amyloid/tau profiles, and higher risk of disease progression [263,266,267]. Nevertheless, results are not consistent: some studies show no correlation between leptin levels, cognition, or disease severity [268], no associations with BMI, weight, or waist circumference [268], and no differences in serum or CSF leptin between AD and controls [269,270,271]. These discrepancies highlight the complexity of leptin biology and suggest that both deficiency and resistance may contribute to disease susceptibility. Animal experiments also confirm these mechanisms and offer insights into the cellular pathways by which leptin may influence AD pathology.

Although leptin transport across the BBB appears intact in the early stages of AD, signaling downstream of ObRb is disrupted, indicating central leptin resistance. Obesity is associated with chronically high leptin levels but impaired hypothalamic and hippocampal leptin receptor signaling [75]. Such “central leptin resistance” combined with reactive peripheral upregulation may be one reason that in obesity, peripheral leptin is unable to exert neuroprotection and may even promote neuroinflammation [75,272]. Reduced ObRb expression has been observed in hippocampal tissues from AD patients, with NFTs sequestering receptors and impairing signaling [270]. In amyloidogenic AD mouse models, neuronal leptin and ObRb expression decline with age, indicating disrupted leptin signaling [269]. Early impairment of adipokine receptor signaling has been documented in APP/PS1 mice, with a marked decline in ObRb expression first observed at 6 months of age. It reduced STAT5B and SOCS1–3 expression detected as early as 3 months, preceding Aβ plaque formation [273].

To test whether systemic leptin alters hippocampal microglia in AD, male APP/PS1 adult (6 months) and aged (12 months) mice received daily intraperitoneal leptin (1 mg/kg) or vehicle for 7 days, followed by quantification of brain Aβ burden and hippocampal microglial activation (Iba-1^+^ cells) and cytokines (IL-1β, IL-6). Leptin reduced Aβ levels in both age groups in comparison to their controls. In adults, leptin increased Iba-1^+^ microglia and elevated IL-1β/IL-6, indicating pro-inflammatory activation. Aged mice displayed higher baseline microglial activation and cytokines than adults, and leptin had no additional effects versus vehicle in this age group. In this study, an age-dependent reduction in ObRb expression was observed in the hippocampus of APP/PS1 mice at 12 months of age. Leptin administration increased hippocampal LepR expression in adult mice; however, this effect was not evident in the aged group. The loss of leptin-induced microglial activation and cytokine response with aging warrants further study [136]. These findings suggest that brain leptin resistance with aging may contribute to the pathophysiology of AD.

As stated above, negative leptin regulators such as SOCS3 and PTP1B, which disrupt JAK2/ObRb signaling, are upregulated in the AD hippocampus [259]. In APP/PS1 mice, early deficits in adipokine receptor signaling are evident at 3 months, with reduced STAT5B and SOCS1–3 expression preceding Aβ plaque formation [273]. Conversely, another study reported that at 18 months, SOCS3 and PTP1B transcripts are upregulated [259]. These findings suggest that in AD, SOCS3 and PTP1B are initially reduced as compensatory adaptations to maintain leptin and cytokine signaling, but become upregulated at later stages, representing maladaptive responses that create a state of central leptin resistance and accelerate neurodegeneration.

In *Tg2576* mice, Aβ can directly impair leptin signaling in the hypothalamus, which exhibits early weight loss and reduced plasma leptin levels before amyloid deposition or cognitive decline. This Aβ-mediated inhibition of hypothalamic neuropeptide Y (NPY) neurons may induce a catabolic state with low leptin, contributing to further neurodegeneration and cognitive decline. The presence of amyloid and tau pathology in the hypothalamus supports a bidirectional relationship, where AD pathology disrupts leptin signaling, and impaired leptin signaling, in turn, accelerates AD progression [267].

Immunohistochemical staining of AD postmortem hippocampal and cortical tissue confirmed the expression of LepR on pyramidal neurons with common co-localization of hyperphosphorylated tau [269,270,274]. In *Tg2576* mice, an amyloidogenic AD model used to study mechanisms of Aβ pathology, neuronal leptin expression is decreased, and ObRb expression declines with age, supporting signaling dysfunction of leptin [269]. Downstream signaling through STAT3 in these tissues was markedly reduced, suggesting a functional deficiency in leptin signaling [269,270,274]. This conforms to the hypothesis that peripheral leptin levels are not sufficient if receptor function or signal transduction within the CNS is impaired. Collectively, these findings suggest that central leptin resistance in AD probably arises from disrupted receptor expression, sequestration by pathological aggregates, and inhibition of downstream pathways by SOCS3 and PTP1B, thereby weakening leptin’s neuroprotective potential. This reduced leptin signaling impairs hippocampal function and weakens neuroprotection against Aβ and tau pathology, which in turn further disrupts neuronal function and leptin transport across the BBB or ChP. Consequently, a vicious cycle develops in which leptin dysfunction and AD pathology reinforce one another, leading to progressive cognitive and metabolic decline.

In a 2018 study using APP/PS1 mice, leptin protein levels and ObR expression were analyzed across disease progression [259]. At 3 and 6 months, leptin protein remained unchanged, while an age-dependent reduction in ObRb occurred in both APP/PS1 and WT mice, more prominently in the neocortex and hippocampus. At this stage, hippocampal ObRb protein was higher in APP/PS1 mice than in WT controls. At 9 months, corresponding to early amyloid pathology, leptin levels were elevated in the hippocampus of APP/PS1 mice but unchanged in the neocortex. By 18 months, leptin levels declined significantly in the hippocampus of APP/PS1 mice and with aging in the neocortex of WT animals, indicating region-specific and age-dependent alterations in leptin signaling. RT-qPCR revealed that hippocampal ObRb mRNA was consistently downregulated in APP/PS1 mice at both 9 and 18 months, despite no changes in neocortical transcript levels. Interestingly, ObRb protein was reduced in the neocortex but increased in the hippocampus of APP/PS1 mice at both ages, suggesting post-transcriptional regulation and regional compensation. Finally, SOCS3 and PTP1B transcript levels were significantly upregulated in the neocortex and hippocampus of APP/PS1 mice at 18 months, but unchanged in WT mice, indicating activation of leptin-related negative feedback pathways during late-stage amyloid pathology, contributing to central leptin resistance despite preserved peripheral levels [259].

Chronic intraperitoneal leptin administration in APP/PS1 double-transgenic mice reduced hippocampal soluble Aβ1-42 by 48% and preserved synaptic density, as evidenced by a 32% higher synaptophysin immunoreactivity compared to saline-treated controls (values quantified via ELISA and immunoblotting). This Aβ reduction is thought to result from leptin’s ability to downregulate BACE1 expression and shift APP processing toward the non-amyloidogenic α-secretase pathway, while simultaneously enhancing microglial phagocytic activity [275]. In an Aβ1-42-induced AD mouse model, intraperitoneal leptin administration improved spatial learning, reduced hippocampal neuronal loss and apoptosis, and suppressed pro-inflammatory cytokine expression. These neuroprotective effects were mediated through activation of the p-Akt signaling pathway [276].

In another study in male APP/PS1 mice, leptin administration enhances adult neurogenesis in both the hippocampal dentate gyrus and the subventricular zone in both adult and aged mice. Chronic leptin treatment promotes neural stem cell (NSC) proliferation and significantly influences the proliferation and differentiation of newborn cells. The long signaling isoform of the leptin receptor, ObRb, was detected in these neurogenic niches by reverse-transcription quantitative PCR and confirmed by immunohistochemistry. Furthermore, leptin modulated astrogliosis, altered microglial cell numbers, and reduced the formation of senile plaques. Notably, leptin attenuated Aβ-induced neurodegeneration and suppressed superoxide anion production [277].

Leptin treatment in *TgCRND8* mice produced robust improvements in AD-like pathology. After eight weeks, leptin significantly reduced brain and serum Aβ1-40 levels, hippocampal amyloid burden, and C99 APP fragments, suggesting β-secretase inhibition. It also lowered tau phosphorylation at AT8 and Ser396 sites without increasing inflammatory markers (CRP, TNF-α, cortisol). These molecular and histopathological changes were accompanied by marked gains in cognitive performance [278]. Leptin-treated mice showed a marked reduction in tau phosphorylation at the pathological Ser396 and Ser404 epitopes. This effect was mediated by PI3K activation, alongside GSK-3β inhibition. These signaling changes suppress tau aggregation and promote microtubule stability. Notably, these molecular effects were accompanied by improvements in hippocampal long-term potentiation and enhanced spatial memory performance [279].

Elevated leptin in leptin resistance affects AD pathogenesis differently from exogenous leptin treatment. In the *PS19* tauopathy mouse model, a high-calorie diet (HCD) induced obesity was associated with marked hyperleptinemia and aggravated both tau pathology and glial activation [280]. Voluntary wheel running normalized circulating leptin and reversed the HCD-induced neuropathological changes. These findings implicate persistent hyperleptinemia as a key driver of tau pathology. Mechanistically, HCD-fed *PS19* mice exhibited reduced nuclear p-STAT3 in hippocampal neurons, indicating central leptin resistance, alongside increased astroglial activation and up-regulation of the short leptin receptor isoform, ObRa, in the hippocampus. In primary astrocyte cultures, leptin up-regulated IL-1β and TNF-α mRNA expression even in the absence of ObRb, suggesting astrocytic leptin hypersensitivity via ObRa. Collectively, these results support a model in which obesity-associated hyperleptinemia promotes astrocytic activation, inflammation, and accelerated tau pathology in *PS19* mice [280]. Collectively, it has been demonstrated that leptin-resistant mice, which develop obesity and a diabetic phenotype, exhibit elevated tau phosphorylation. In contrast, in vitro exposure of neuronal cells to leptin reduced tau phosphorylation. Researchers transduced leptin-receptor-deficient diabetic mice with the mutant human tau (P301L) using an adeno-associated virus (AAV). These mice displayed a marked increase in hippocampal tau phosphorylation and a higher burden of NFTs compared with controls. Collectively, these findings indicate that leptin resistance-associated obesity and diabetes can accelerate tau pathology in vivo [281] (Figure 3).

These preclinical findings align closely with mechanisms inferred from human studies, supporting the notion that leptin modulates both Aβ and tau pathology in ways that may influence disease progression.

### 6.3. Leptin Levels Across AD

The most compelling human evidence for leptin’s anti-AD action is from large, well-characterized longitudinal cohorts with biomarker and cognitive follow-up. The Framingham study recruited 785 cognitively intact older subjects (mean age 79 ± 5 years; 62% females) with fasting plasma leptin measured by radioimmunoassay [10]. After sex-standardization, each 1-SD increase in leptin was associated with hazard ratios of 0.68 for all-cause dementia and 0.60 for AD during a median follow-up period of 8.3 years, after adjusting for age, waist-hip ratio, BMI, systolic blood pressure, antihypertensive medication, diabetes, smoking, cholesterol, homocysteine, and APOE ε4 status [10]. 12-year cumulative incidence was about 25% in the lowest quartile of leptin and about 6% in the highest quartile. Of particular interest, this protective association was significant only among individuals with BMI < 30 kg/m^2^, suggesting that central leptin resistance in obesity may impair these effects [10].

A Dutch population-based prospective cohort study (Rotterdam study) evaluated 1165 dementia-free adults aged 55–80 years. Serum leptin levels from 1997–1999 were measured using ELISA, and participants were followed until 2018. After adjusting for BMI, waist circumference, homeostatic model assessment for insulin resistance (HOMA-IR), hypertension, lipid levels, and APOE ε4 status, individuals in the highest sex-specific tertile of leptin had a lower risk of developing dementia and AD. Unexpectedly, this association was stronger at higher BMI, possibly due to weight loss preceding dementia (masking leptin’s protection) and greater leptin variability in obesity, which may explain the inconsistency with prior findings [282].

In the Texas Alzheimer’s Research and Care Consortium (TARCC) study, eight adipokines, including leptin, were analyzed as a combined “adipokine” construct using structural equation modeling to examine their relationship with dementia severity. This composite explained a substantial portion of dementia-related variance, and its scores rose progressively from cognitively normal individuals to those with mild cognitive impairment (MCI) and AD. The adipokine composite also showed strong diagnostic accuracy in distinguishing AD, MCI, and controls. These findings suggest that peripheral adipokines, including leptin, are closely linked to dementia severity and could serve as reproducible blood-based biomarkers of cognitive decline [11]. In another study, baseline measurements also demonstrated reduced plasma leptin and leptin/BMI ratio in MCI and AD subjects compared to normal controls [266].

Similarly, the Health and Body Composition (ABC) study followed over 3000 older adults (baseline age 70–79 years, 48% Black, 52% White) through trajectories of cognitive decline [283]. Baseline fasting plasma leptin was quantified by ELISA. In models controlling for BMI, percentage body fat, diabetes, cardiovascular disease, C-reactive protein, IL-6, and physical activity, subjects in the highest quintile of leptin declined less over four years on the modified mini-mental state examination (MMSE) [283]. Stratified analyses showed the benefit was mostly limited to normal-weight individuals, while these associations were diminished or not observed in overweight/obese individuals [283].

These associations were most pronounced in non-obese females, in line with earlier findings of possible sex-hormone modulation and that obesity-related central leptin resistance impairs its beneficial effects. In the prospective study of osteoporotic fractures, 579 older females (mean age 82.6 years) who were dementia-free at baseline were followed for incident dementia or MCI. Serum leptin was measured from baseline. A significant association was found between log-transformed leptin and BMI category. Among females with BMI < 25 kg/m^2^, each 1-SD increase in log leptin (0.91 ng/mL) was associated with 32% reduction odds of dementia or MCI. This association was not observed in females with BMI ≥ 25 kg/m [284].

In a case–control study, researchers compared 40 patients with mild MCI to 40 age-, sex-, and body fat-matched healthy controls to investigate the relationship between leptin, memory performance, and hippocampal integrity. The MCI patients had significantly lower serum leptin levels than controls, independent of sex, age, body fat, and metabolic factors. They also showed poorer memory performance and reduced hippocampal volume and microstructural integrity. While leptin was not directly associated with memory scores, mediation analyses indicated that lower leptin contributed to poorer memory indirectly through its adverse effects on right hippocampal volume and left hippocampal microstructure. These findings suggest that reduced leptin may promote hippocampal degeneration and memory decline in MCI, independent of metabolic confounders [285].

In a recent cross-sectional study of 148 AD patients and 110 cognitively normal controls, lower plasma leptin levels were associated with higher odds of AD (OR = 0.417, 95% CI: 0.272–0.638) after adjustment for conventional risk factors. Across all participants, plasma leptin was positively correlated with MMSE scores and negatively with clinical dementia rating (CDR) scores. These findings suggest that reduced leptin is linked to AD and the severity of symptoms [286].

Some studies have reported that greater adiposity and leptin in older adults are linked to a lower risk of AD-related cognitive decline. In Alzheimer’s disease neuroimaging initiative (ADNI), amongst 716 older adults with amnestic MCI, higher baseline BMI was associated with greater cerebral metabolic rate of glucose (rCMRgl) and slower rCMRgl decline in AD-vulnerable regions over an average 3.3-year follow-up. However, analyses of CSF and plasma samples showed that leptin concentrations were instead linked to lower baseline rCMRgl in mesocorticolimbic regions involved in energy homeostasis, and did not explain the BMI-related protective association [287].

In the BAriatric surgery Rijnstate and Radboudumc neuroImaging and Cognition in Obesity (BARICO) cohort study of individuals with severe obesity undergoing Roux-en-Y gastric bypass, substantial postoperative weight loss was accompanied by decreased plasma leptin. Cognitive improvement occurred in 43.8% of participants. It was specifically associated with lower postoperative leptin and CRP levels, suggesting that reductions in leptin in obese patients with potential leptin resistance may even partly mediate the cognitive benefits of bariatric surgery [288].

In a Kansas University Medical Center (KUMC) cohort comparing nonobese AD patients and cognitively unimpaired controls, plasma leptin levels were reduced in male AD patients but unchanged in females. Leptin showed no association with cognitive performance or circulating AD biomarkers in either sex. Interestingly, in females but not males, plasma leptin correlated with BMI, HDL-cholesterol, and lipid ratios. These findings suggest that leptin deficiency may characterize nonobese male AD patients, reflecting systemic metabolic disturbances, while plasma leptin does not appear to reflect disease severity or progression [289].

Not all studies have demonstrated a positive correlation between leptin and AD. For instance, one investigation reported no association between leptin levels and cognition or disease severity in AD patients and further found no correlations between leptin levels and BMI, body weight, or waist circumference [268]. Similarly, a study in adult patients with a mean age of 60 years with AD or vascular dementia showed serum leptin levels comparable to those of healthy controls and individuals with subjective memory complaints [271]. In a cohort including patients with AD, vascular dementia, and mixed dementia, alongside individuals with MCI and healthy controls, serum leptin did not differ between dementia patients and controls, indicating no evident association of leptin with dementia presence or subtype in this study [290]. In another study comparing 26 AD, 21 frontotemporal dementia (FTD), and 23 control participants, no differences in leptin levels were observed between cases and controls after adjusting for sex, age, BMI, HOMA-IR, and other adipokines. However, female patients with AD showed significantly higher leptin levels than female controls [291]. In MCI participants, fasting leptin did not predict 3-year dementia risk after adjustment for BMI, hypertension, insulin, and C-reactive protein [292]. Similarly, midlife leptin was not related to dementia over 32 years, suggesting that alterations in circulating leptin levels associated with AD may emerge in late life but not during midlife [293].

Despite variability across individual studies, pooled evidence supports a protective inverse association between leptin level and AD. A 2021 meta-analysis of 14 case–control and cohort studies (total ≈ 6800) reported that elevated circulating leptin was associated with a pooled relative risk for AD of 0.68 (95% CI: 0.54–0.85), with more potent effects in prospective versus cross-sectional designs [294]. Heterogeneity was moderate (I^2^ = 51%), in large part due to adjustment for BMI status and fasting status [294].

Methodological challenges remain in leptin-AD research. As leptin secretion is strongly correlated with adiposity, failure to control for BMI or fat mass can create confounding. Large prospective cohorts such as Framingham, Health ABC, and Rotterdam have controlled these factors, and protective associations persist [10,283,295]. In obesity, elevated leptin levels are not protective, consistent with central leptin resistance [75]. Controlling for BMI, females exhibit higher leptin than males; however, the direction and extent of sex-specific effect modification vary across cohorts [10,283].

### 6.4. Leptin as an Imaging and CSF Biomarker for AD

Besides epidemiologic data, neuroimaging studies provided converging structural and functional evidence implicating leptin in brain integrity in aging and AD. Cross-sectional MRIs on 198 dementia-free participants of the Framingham cohort revealed that plasma leptin was associated with greater total cerebral brain volume and lower lateral ventricular volume following adjustment for age, sex, education, vascular risk factors, and BMI [10]. In fact, for each 1-SD increase in leptin, there was 0.8% larger brain volume and 5% smaller lateral ventricular volume [10].

Rare congenital leptin deficiency gives further insights into the neuroanatomical actions of leptin. Patients with leptin deficiency were treated in an open-label study, with once-daily subcutaneous synthetic, injectable version of leptin. MRI voxel-based morphometry demonstrated significant gray matter volume increases in the anterior cingulate cortex, inferior parietal lobule, and cerebellum, and improved memory test performance [296]. Although not AD-specific, these reports constitute direct proof-of-concept that leptin can promote structural and functional cerebral change in humans.

Preclinical high-resolution biomarker imaging in the Korean Brain Aging Study for the Early Diagnosis and Prediction of Alzheimer’s disease (KBASE) has extended these observations to preclinical phases. In 208 healthy older adults (mean age 66.0 ± 11.3 years; 54.8% female; 17.8% APOE ε4 carriers), fasting plasma leptin by ELISA was inversely associated with cortical Aβ burden by carbon-11 Pittsburgh compound B (^11^C-PiB) positron emission tomography (PET). In models adjusted for age, sex, education, BMI, vascular risk, and APOE ε4, each 1-ng/mL increase in leptin was related to a β coefficient of −0.04 for cortical standardized uptake value ratios (SUVRs), a quantitative measure of Aβ deposition in the brain’s cortex [297]. Long-term follow-up for 2 years revealed that low baseline leptin predicted greater tau accumulation on ^18^F-AV1451 PET (β = −0.06; 95% CI: −0.11 to −0.01) [297].

Biomarker research also demonstrated CSF leptin and AD pathology associations. In one series of 1036 memory clinic patients in whom both CSF and plasma sampling were undertaken, low plasma leptin was associated with CSF amyloid positivity (Aβ1-42 < 500 pg/mL), especially in those who were both amyloid- and tau-positive [298]. Multivariable regression, adjusting for age, sex, BMI, and APOE ε4 status, showed that a decrease in plasma leptin was associated with decreased CSF Aβ1-42 and increased CSF phosphorylated tau181 [298]. Clinically, AD patients had a 7.3 ng/mL lower plasma leptin level on average than non-AD cognitive impairment [298].

In a cross-sectional study of 140 cognitively impaired patients, CSF and plasma leptin levels were strongly correlated (r = 0.79). Individuals with an AD biomarker-positive CSF profile had significantly lower CSF leptin concentrations than neurological controls, and CSF leptin remained inversely associated with the Aβ ratio after adjusting for age, sex, BMI, and estimated glomerular filtration rate (eGFR). These findings indicate that CSF leptin is reduced in AD, correlates closely with plasma leptin, and is linked to brain amyloid pathology, suggesting that plasma leptin may serve as a peripheral indicator of central leptin activity [299].

A recent study examined how leptin links adiposity-related inflammation to neuropsychiatric symptoms (NPS) in AD patients. Plasma leptin levels correlated with BMI and predicted increased TSPO-PET signal (^11^C-DPA-713-BPND) in the insula, which was also associated with higher neuropsychiatric inventory questionnaire (NPI-Q) scores. The model supported leptin as a mediator between adiposity and NPS via insular neuroinflammation [300].

However, another study found no significant differences in CSF leptin concentrations between AD patients and controls [269]. As previously stated, the development of neuronal leptin resistance in AD may account for the conflicting reports regarding unchanged or increased CSF leptin levels. Another study examined the relationship between plasma leptin levels, cognitive performance, and brain structure. After adjusting for BMI, APOE4 status, and other clinical factors, no associations were found between leptin and any cognitive domains or MRI brain volume measures. However, BMI significantly modified the association between leptin and verbal memory performance. In individuals with normal BMI, higher circulating leptin concentrations were positively associated with verbal memory scores, whereas this relationship was not significant in overweight and obese participants [301]. In a longitudinal ADNI study of individuals with MCI, plasma leptin levels were not associated with future cognitive decline or cortical thinning, regardless of Aβ status [302].

### 6.5. Genetics in the Context of Leptin-Mediated Risk in AD

In 2010, a study examined a *LEPR* polymorphism involving a glutamine-to-arginine substitution at codon 223 (Gln223Arg) in a Japanese population with late-onset AD (LOAD) and reported no significant association with disease risk [303]. Similarly, a Mendelian randomization analysis using SNPs associated with circulating leptin and soluble LepR levels found no significant link between genetically predicted leptin signaling and AD susceptibility [304]. In contrast, another study suggested that the *LEP* variant rs10258300 may exert a protective effect against neurodegenerative dysfunction [305]. More recently, a large-scale Mendelian randomization study using genome-wide association data demonstrated that genetically predicted higher leptin levels were significantly associated with a reduced risk of AD (OR 0.838, 95% CI 0.741–0.948, *p* = 0.005), whereas soluble LepR levels showed no significant association; notably, this protective effect was attenuated after adjustment for insulin sensitivity index (ISI) but remained independent of body mass index (BMI), with no evidence of reverse causation, pleiotropy, or heterogeneity observed, supporting the robustness of the findings [306].

### 6.6. Therapeutic Implications of Leptin in AD and Future Directions

The timing of leptin measurement is critical. Midlife or early late-life measurements, well before AD symptom onset, yield the strongest leptin protective associations [295,307]. By contrast, leptin levels in symptomatic AD are often reduced, reflecting disease-associated weight loss, adipose reduction, or altered hypothalamic regulation, which in turn can lower leptin levels and exacerbate pathological processes in AD. This biphasic pattern mirrors leptin’s relationship to dementia risk: protective in midlife, but often reversed or normal in late life. Generally, evidence from epidemiology, neuroimaging, and biomarker studies suggests that sustained normal leptin signaling from midlife may reduce AD risk by limiting amyloid and tau pathology, preserving synapses, and reducing neuroinflammation [308].

Both central leptin resistance and leptin declines share similar pathway of cognitive impairment. Longitudinal studies need to link leptin levels with amyloid/tau PET, CSF biomarkers, APOE genotypes, and measures of adiposity, ideally from midlife onwards. Leptin assay conditions (fasting or non-fasting, serum or plasma, ELISA or RIA) require standardization for comparison. Given its dual role as an immune modulator and metabolic regulator, leptin remains a promising candidate for both biomarker development and therapeutic targeting in neurodegenerative diseases.

As stated earlier, GLP-1RAs improve weight loss and decreases leptin serum levels. A phase 3 trial of the GLP-1RA, semaglutide, is currently underway in early-stage symptomatic AD to monitor its effects on disease biomarkers and clinical outcomes [309]. In a study assessing the comparative effectiveness of GLP-1RAs and metformin in reducing dementia risk among individuals with type 2 diabetes, GLP-1RAs reduced dementia risk. Leptin resistance was not discussed; however, as only 4% of participants were of normal weight and others were overweight or obese, it can be assumed that they had some extent of leptin resistance. GLP-1RAs may therefore reduce leptin levels and leptin resistance, potentially contributing to the observed reduction in dementia risk [310]. More research is needed to consider the roles of BMI and leptin resistance in the context of GLP-1 receptor agonist therapy in AD patients or in reducing risk of AD.

## 7. Conclusions

Leptin signaling regulates both innate and adaptive immunity. At physiologic levels, leptin exerts neuroprotective effects, namely enhancing synaptic integrity, promoting Aβ clearance, and decreasing tau phosphorylation in diseases like AD [10]; leptin levels are reduced among AD patients with normal BMI [286,295]. Leptin also potentiates microglial activation, promotes DC maturation, enhances neutrophil migration, and stimulates T cell proliferation biased towards Th1 and Th17 polarization, while suppressing Tregs [115]; promoting the proinflammatory environment that may underlie MS pathogenesis. Leptin levels are elevated in both MS patients and in EAE models, where higher concentrations correlate with relapse rates, disability progression, and EDSS scores, suggesting a potential role in driving autoimmunity [192,213]. These dual and context-dependent functions underscore how leptin’s impact relates to disease state, patient phenotype, and circulating leptin levels.

Obesity acts as a confounding factor, not only serving as a potential risk factor for neuroinflammatory diseases such as MS and AD but also inducing leptin resistance through which metabolic dysfunction drives immune dysregulation and neuroinflammation [66]. Leptin resistance, characterized by reduced CNS sensitivity and impaired leptin signaling, leads to elevated peripheral leptin levels that may hypothetically promote inflammation and contribute to MS pathogenesis, while simultaneously diminishing leptin’s central responsiveness and neuroprotective effects contribute to AD. GLP-1RA treatments, which indirectly reduce leptin levels [239], have been shown to improve clinical outcomes in EAE and to reduce the risk of MS development [240,241,242,243,244,245]. A phase 3 trial of GLP-1RA is currently evaluating its effects on biomarkers and clinical outcomes in early-stage AD [309]. In patients with type 2 diabetes, GLP-1RAs have been associated with reduced dementia risk. Since most participants in the trial were overweight or obese, leptin resistance was likely present, suggesting that the observed cognitive benefits may partly reflect improved leptin sensitivity [310]. However, its effects in conditions without leptin resistance remain to be elucidated. Future studies should investigate the effects of leptin reduction across different BMI categories and consider the potential presence of leptin resistance to account for these differences.

Conceivably, therapeutic measures for MS, focusing on obesity-related mechanisms, could be envisioned. Based on what is discussed here, leptin exhibits opposing functions; complete antagonism may be detrimental in non-obese individuals since basal leptin levels are essential for energy homeostasis and immune modulation. Noticeably, as a pro-inflammatory and neuroprotective signaling molecule, leptin reduction should be weighed against the pathological hyperleptinemia in neuroinflammatory conditions such as MS, as opposed to its protective effects in chronic neurodegenerative diseases such as AD.

## Data Availability

No new data were created or analyzed in this study. Data sharing is not applicable to this article.
